# TBX3 promotes progression of pre‐invasive breast cancer cells by inducing EMT and directly up‐regulating SLUG

**DOI:** 10.1002/path.5245

**Published:** 2019-04-08

**Authors:** Milica Krstic, Bart Kolendowski, Matthew J Cecchini, Carl O Postenka, Haider M Hassan, Joseph Andrews, Connor D MacMillan, Karla C Williams, Hon S Leong, Muriel Brackstone, Joseph Torchia, Ann F Chambers, Alan B Tuck

**Affiliations:** ^1^ Department of Oncology, Schulich School of Medicine and Dentistry Western University London ON Canada; ^2^ The Pamela Greenaway‐Kohlmeier Translational Breast Cancer Research Unit, London Regional Cancer Program London Health Sciences Centre London ON Canada; ^3^ Department of Pathology, Schulich School of Medicine and Dentistry Western University London ON Canada; ^4^ Department of Biochemistry, Schulich School of Medicine and Dentistry Western University London ON Canada; ^5^ Mary & John Knight Translational Ovarian Cancer Research Unit, London Regional Cancer Program London Health Sciences Centre London ON Canada; ^6^ Faculty of Medicine University of British Columbia Vancouver BC Canada; ^7^ Faculty of Pharmaceutical Sciences University of British Columbia Vancouver BC Canada; ^8^ Departments of Urology Physiology and Biomedical Engineering, Mayo Clinic Rochester MN USA

**Keywords:** breast cancer, TBX3, SLUG, ductal carcinoma *in situ* (DCIS), epithelial‐to‐mesenchymal transition (EMT)

## Abstract

The acquisition of cellular invasiveness by breast epithelial cells and subsequent transition from ductal carcinoma *in situ* (DCIS) to invasive breast cancer is a critical step in breast cancer progression. Little is known about the molecular dynamics governing this transition. We have previously shown that overexpression of the transcriptional regulator TBX3 in DCIS‐like cells increases survival, growth, and invasiveness. To explore this mechanism further and assess direct transcriptional targets of TBX3 in a high‐resolution, isoform‐specific context, we conducted genome‐wide chromatin‐immunoprecipitation (ChIP) arrays coupled with transcriptomic analysis. We show that TBX3 regulates several epithelial–mesenchymal transition (EMT)‐related genes, including *SLUG* and *TWIST1*. Importantly, we demonstrate that TBX3 is a direct regulator of *SLUG* expression, and *SLUG* expression is required for TBX3‐induced migration and invasion. Assessing TBX3 by immunohistochemistry in early‐stage (stage 0 and stage I) breast cancers revealed high expression in low‐grade lesions. Within a second independent early‐stage non‐high‐grade cohort, we observed an association between TBX3 level in the DCIS and size of the invasive focus. Additionally, there was a positive correlation between TBX3 and SLUG, and TBX3 and TWIST1 in the invasive carcinoma. Pathway analysis revealed altered expression of several proteases and their inhibitors, consistent with the ability to degrade basement membrane *in vivo*. These findings strongly suggest the involvement of TBX3 in the promotion of invasiveness and progression of early‐stage pre‐invasive breast cancer to invasive carcinoma through the low‐grade molecular pathway. © 2019 The Authors. *The Journal of Pathology* published by John Wiley & Sons Ltd on behalf of Pathological Society of Great Britain and Ireland.

## Introduction

Tumorigenesis within the breast is thought to involve advancement through distinct stages, from benign ductal epithelial hyperplasia to atypical ductal hyperplasia (ADH), ductal carcinoma *in situ* (DCIS), and, ultimately, invasive and metastatic carcinoma 1. It is estimated that approximately 25–50% of DCIS will progress to invasive carcinoma during the lifetime of the patient, generating potentially life‐threatening disease 2. Clinical management of patients with DCIS usually consists of surgical resection with additional radiation therapy for most patients 3, 4. Several recent studies, however, have suggested that a subset of patients with early‐stage (non‐high‐grade) DCIS do not benefit from either surgery alone or combined surgery and radiation, raising concern regarding overtreatment of this subpopulation 2, 5, 6. Paradoxically, we are currently limited in our ability to identify patients with DCIS who have an intrinsically higher risk of local and invasive recurrence, such that there is also a risk of undertreatment if excision is incomplete or radiation therapy is omitted.

The 21T series cell lines have been proposed as a unique experimental model of breast cancer progression 7. Three cell lines were isolated from a single patient and stably represent distinct stages of progression: 21PT cells mimic ADH (non‐tumorigenic); 21NT cells mimic DCIS (tumorigenic and non‐metastatic); and 21MT‐1 cells mimic invasive mammary carcinoma (tumorigenic and metastatic) in xenograft experiments 7, 8. We have conducted extensive characterization of these cell lines 8. Importantly, we found that during the transition from the clonally related DCIS‐like 21NT cells to invasive 21MT‐1 cells, TBX3 was within the list of clinically relevant up‐regulated transcripts 8.

TBX3 is a member of the T‐box family of transcription factors involved in development 9. TBX3 levels are up‐regulated in several cancers, with most of the literature focusing on breast cancer 8, 10, 11, 12 and melanoma 13, 14. Due to alternative splicing, two TBX3 isoforms exist: TBX3iso1 and TBX3iso2. TBX3iso2 contains an additional 20‐amino acid sequence in the DNA binding domain attributed to the 2a exon, which TBX3iso1 lacks. Differing expression of TBX3 isoforms has been reported in several breast cancer cell lines 12 and there is controversy as to whether the two isoforms have unique functions 12, 15, 16. Accumulating evidence suggests that TBX3‐mediated transcriptional repression of p14^ARF^ 11, 17 and/or p21^CIP1^ 16, 18 plays a role in driving cancer progression through bypassing cellular senescence. Recent work has begun to identify a link between TBX3 and epithelial‐to‐mesenchymal transition (EMT) 19, with direct down‐regulation of E‐cadherin 13. These findings are important, as an EMT phenotype has been associated with the acquisition of migratory and invasive properties, suppression of senescence and apoptosis, and therapeutic resistance 20. SLUG (encoded by the *SNAI2* gene) is a member of the SNAIL family of transcription factors and a key mediator in the promotion of EMT 20, 21. Specifically, SLUG has been shown to trigger the initial phases of the EMT process 22.

In this study, we explored the role of TBX3 in breast cancer progression pathways, focusing specifically on its involvement in the induction of EMT and the transition from DCIS to invasive carcinoma. Immunohistochemical analysis revealed that TBX3 levels are elevated in low‐grade, pre‐invasive DCIS with an associated invasive component, and significantly correlated with the size of the invasive focus. Using genome‐wide bioinformatic approaches along with more conventional *in vitro* studies, we have identified *SLUG* and *TWIST1* as downstream targets of TBX3 and have assessed their levels by immunohistochemistry in two independent patient cohorts. Our findings suggest that TBX3 facilitates the process of early invasion in DCIS by promoting the induction of EMT and tumor progression through the low‐grade pathway, as described by Bombonati and Sgroi 23. Finally, we propose a progression model in which SLUG is an important and necessary effector downstream of TBX3, leading to increased motility and induction of key invasiveness‐associated genes.

## Materials and methods

### Cell lines and culture conditions

The 21T series cell lines (21NT and 21MT‐1) were obtained as a gift from Dr Vimla Band 7. 21T series cell lines underwent authentication testing by IDEXX Radil (IDEXX Bioanalytics, Missouri, CA, USA; Case No 20250‐2013). Cells were maintained in αMEM media supplemented with 2 mm l‐glutamine, 1 μg/ml insulin, 12.5 ng/ml EGF, 2.8 μm hydrocortisone, 10 mm HEPES, 1 mm sodium pyruvate, 0.1 mm non‐essential amino acids, 50 μg/ml gentamycin sulfate, and 10% FBS (αHE10F). All reagents were obtained from Wisent Inc (Saint‐Jean‐Baptiste, QC, Canada). Stable 21NT transfectants previously generated 24 were maintained in αHE10F medium supplemented with 500 μg/ml G418 as a selection marker. Stable lentiviral transductants were maintained in αHE10F medium supplemented with either 0.8 μg/ml puromycin (TBX3 knockdown lines) or 500 μm hygromycin (SLUG knockdown lines) as a selection marker. Details related to the generation of cell lines are located in the supplementary material, Supplementary materials and methods.

Cell lines representing other molecular breast cancer subtypes were purchased from ATCC (Manassas, VA, USA). Cell lines were maintained in their respective media: T47D (RPMI), SKBR3 (McCoy's 5A), and MDA‐MB‐468 (αMEM), each supplemented with 10% FBS.

### *In vitro* and *in vivo* characterization of cell lines

Detailed protocols related to western blots, immunofluorescence staining, migration assays, Transwell invasion assays, chick choriallantoic membrane (CAM) assays, gelatin zymography, cell–cell adhesion assay, and *in vitro* gelatin degradation assays 25 are located in the supplementary material, Supplementary materials and methods.

### Genomic and transcriptomic analyses

Detailed protocols related to qRT‐PCR, chromatin immunoprecipitation (ChIP), RNA‐Seq (GEO Accession Number: GSE126153), ChIP promoter arrays (GEO Accession Number: GSE126154), and the bioinformatics analyses 26 may be found in the supplementary material, Supplementary materials and methods.

### Patient characteristics and immunohistochemistry study

Patients with early‐stage breast cancer were identified from the London Breast Cancer Database on the basis of having either DCIS only (stage 0) or DCIS with an associated invasive component (stage I; ≤ 2 cm, and either pN0 or pN0mi). Cohort 1 consisted of 186 patients with low‐, intermediate‐ or high‐grade DCIS with no invasion or early invasion (stage 0 or stage I). Cohort 2 consisted of 118 patients with non‐high‐grade DCIS with the aforementioned characteristics, with either no invasion or early invasion (stage 0 or stage I). Clinicopathological variables for cohort 1 and cohort 2 patients entered into this study are listed in Table 1. There was no overlap between patients in cohort 1 and cohort 2. The study was conducted under a protocol approved by the Western University Health Sciences Research Ethics Board and the Clinical Research Impact Committee of Lawson Health Research Institute. Immunohistochemical staining was conducted using the following antibodies: TBX3 (Abcam, Cambridge, MA, USA; ab99302; 1/200), SLUG (Abcam; ab27568; 1/750), and TWIST1 (Abcam; ab50887; 1/750). Details relating to immunohistochemistry protocol and quantification methods are located in the supplementary material, Supplementary materials and methods.

**Table 1:** Clinicopathologic variables for patients entered into Cohort 1 and Cohort 2

	Cohort 1	Cohort 2
Characteristic	No of patients (%)	No of patients (%)
Total	186 (100)	118 (100)
Age (years)		
≤ 50	17 (9.1)	14 (11.9)
> 50	169 (90.9)	104 (88.1)
Associated invasion		
No invasion	100 (53.8)	84 (71.2)
Micro‐invasion	12 (6.5)	2 (1.7)
Invasion	74 (39.8)	32 (27.1)
Histological type of invasive cancer		
Total cases with invasion	74 (100)	34 (100)
NST	55 (74.3)	26 (76.5)
NST with lobular features	9 (12.2)	3 (8.8)
NST with tubular features	3 (4.1)	3 (8.8)
NST with mucinous features	3 (4.1)	0 (0.0)
NST with micropapillary features	1 (1.4)	0 (0.0)
Invasive lobular carcinoma	1 (1.4)	0 (0.0)
Invasive tubular carcinoma	1 (1.4)	1 (2.9)
Invasive mucinous carcinoma	1 (1.4)	1 (2.9)
IMC histologic grade		
Total cases with invasion	74 (100)	34 (100)
Low	26 (35.1)	16 (47.1)
Intermediate	36 (48.6)	18 (52.9)
High	12 (16.2)	0 (0.0)
DCIS nuclear grade		
Total cases with DCIS	100 (100)	118 (100)
Low	10 (10.0)	16 (13.6)
Low + intermediate	0 (0.0)	44 (37.3)
Intermediate	41 (41.0)	58 (49.2)
High	49 (49.0)	0 (0.0)
Hormone receptor status*		
ER‐positive	66/82 (80.5)	33/34 (97.1)
PR‐positive	52/71 (73.2)	29/34 (85.3)
HER2‐positive	16/57 (28.1)	3/34 (8.8)
Recurrence		
No recurrence	162 (87.1)	
Yes recurrence	13 (7.0)	
Invasive recurrence	10 (76.9)	
Non‐invasive recurrence	3 (23.1)	
Unknown	11 (5.9)	118/118 (100)
Micro‐metastasis	8 (4.3)	0 (0.0)

NST, no special type; IMC, invasive mammary carcinoma; DCIS, ductal carcinoma *in situ*; ER, estrogen receptor; PR, progesterone receptor; HER2, human epidermal growth factor receptor 2.

* Only available for patients with IMC.

## Results

### TBX3 overexpression is associated with an invasive and EMT phenotype

**Figure 1:**
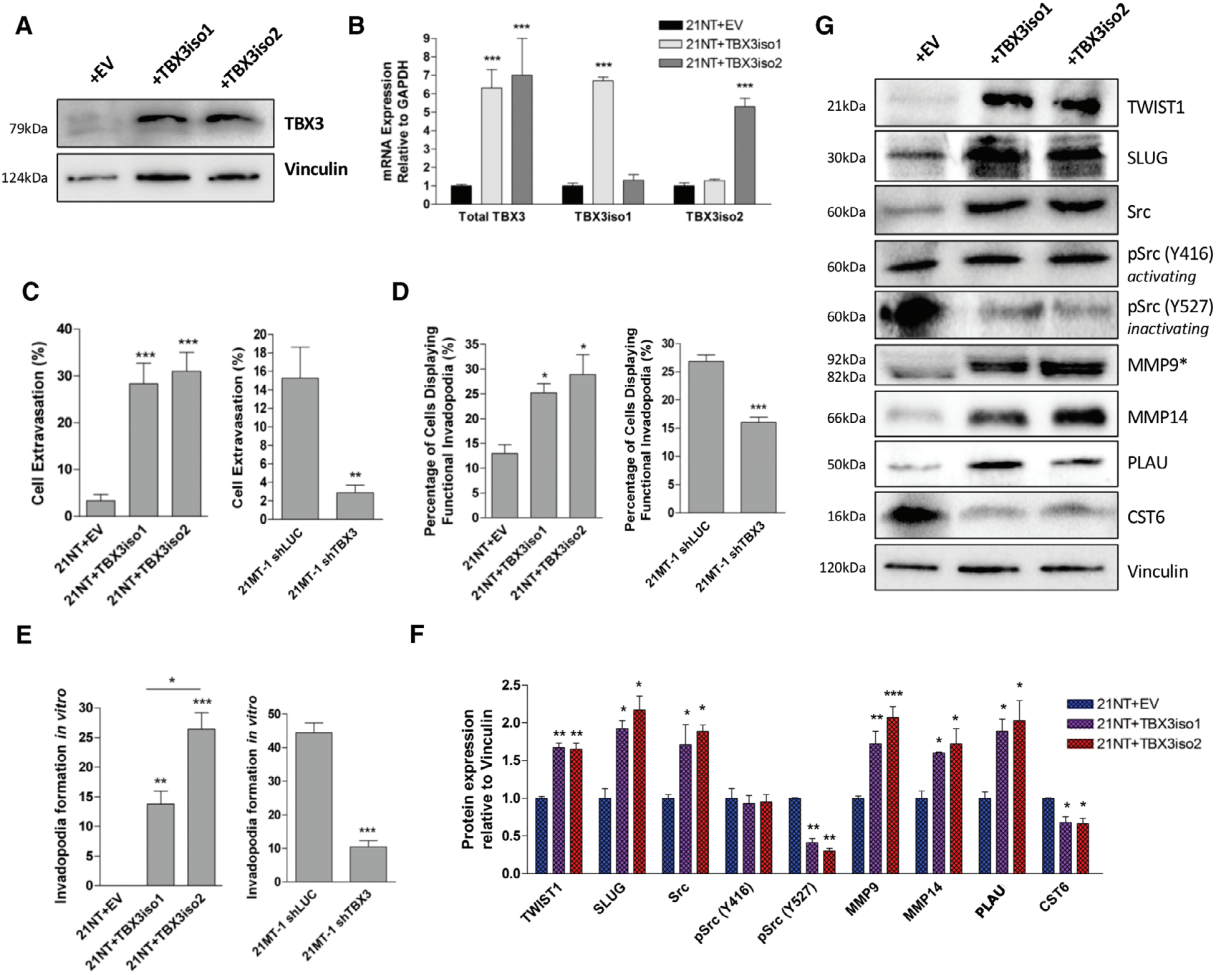
TBX3 overexpression is associated with an invasive and EMT phenotype. (A) Total TBX3 protein levels were assessed by western blot in 21NT transfectant cells. (B) Total *TBX3*, *TBX3iso1*, and *TBX3iso2* transcript levels were assessed by RT‐qPCR in 21NT transfectant cells, normalized to *GAPDH* levels, and depicted as fold change relative to the empty vector control. Means derived from three biological replicates were used during analysis. (C, D) Assessment of cell extravasation and functional invadopodia formation *in vivo* in the chick chorioallantoic membrane (CAM) model. Green (CMFDA) fluorescently labeled cells were injected into the vasculature of the CAM (50 000 cells each), and the vasculature was labeled red with lectin‐rhodamine at *T* = 0. For cell extravasation, cells were counted 24 h post‐injection in a 1 cm^2^ area across 30 biological replicates. For functional invadopodia formation, a binary quantification of *in vivo* invadopodia formation was conducted 6 h post‐cell injection. Cells were deemed positive for invadopodia formation when they possessed protrusions from the vasculature into the stroma. Values reported represent the percentage of cells displaying functional invadopodia *in vivo* across six biological replicates consisting of approximately 30 scanned cells each. (E) *In vitro* invadopodia formation assay for assessment of local cell invasion. Cells were added to red fluorescently labeled gelatin and incubated for 12–24 h (as optimized) to allow for substrate degradation. Cells were visualized using labelled phalloidin for identification of cellular F‐actin cores at sites of local matrix degradation, signifying the presence of invadopodia. Percentage of cells forming invadopodia was quantified from four biological replicates, assessing invadopodia formation in 10–20 random, non‐overlapping fields. (F, G) Protein levels of EMT markers by western blot. Protein samples were separated by 10% SDS‐PAGE and quantified by densitometry. Protein levels were normalized to vinculin, which served as the loading control for all proteins aside from MMP9 (*secreted protein); in the latter case, MMP9 levels in the conditioned media samples were normalized to total protein per lane based on Ponceau staining of the membrane. Where quantification of western blots is shown, data were acquired through densitometric quantifications across three biological replicates. *p < 0.05; **p < 0.01; ***p < 0.001 by one‐way ANOVA with Tukey's *post hoc* test for comparison between three groups, and Student's *t*‐test for comparison between two groups. Error bars represent standard deviation.

To examine the role of TBX3 in breast cancer, non‐invasive 21NT cells (which endogenously express low levels of TBX3) were stably transfected with either an empty vector, TBX3iso1 or TBX3iso2. Structural differences between TBX3 isoforms are shown in the supplementary material, Figure S1. Isoform specific up‐regulation was confirmed at the mRNA level using isoform‐specific primers, in addition to up‐regulation of total TBX3 protein levels (Figure 1A,B and supplementary material, Figure S2A). Functionally, up‐regulation of either TBX3 isoform led to an increase in migration (supplementary material, Figure S3A). TBX3‐overexpressing cells also exhibited reduced cell‐to‐cell adhesion *in vitro* (supplementary material, Figure S3B). In the presence of calcium ions (+ CaCl_2_) or after adding increasing doses of EDTA to chelate free calcium, there were reduced levels of cell‐to‐cell adhesion, implying that several cell‐adhesion molecules may be involved (not solely Ca^2+^‐dependent cadherins). Loss of E‐cadherin has been associated with the switch to an EMT phenotype 23. Additionally, TBX3 is able to directly down‐regulate E‐cadherin in melanoma lines 13. We confirmed a down‐regulation of E‐cadherin protein levels by immunofluorescence, as well as decreased membrane localization with TBX3 overexpression (supplementary material, Figure S3C).

In order to assess the aggressiveness of the cell lines in an *in vivo* system, we employed the chick embryo chorioallantoic membrane (CAM) model. Fluorescently labeled cells were injected into the vasculature of chicks at embryonic day 12 and cell extravasation was assessed as previously described 27 (Figure 1C). Overexpression of either TBX3 isoform in 21NT cells resulted in an increase in extravasation within the CAM. Extravasation rates were drastically reduced with shRNA‐mediated knockdown of total *TBX3* in invasive 21MT‐1 cells, which natively express TBX3 at high levels (levels shown in supplementary material, Figure S2B–D). To confirm that the cells are better able to exit the vasculature (and are not simply better able to survive at *T* = 24 h), we performed live functional invadopodia assays within the CAM using confocal microscopy. Six hours after injecting fluorescently labeled cells into the CAM vasculature, a binary quantification of invadopodia formation was conducted (Figure 1D). An increase in functional invadopodia was observed with overexpression of TBX3 in 21NT cells. Furthermore, the presence of functional invadopodia was reduced with TBX3 knockdown in 21MT‐1 cells. Representative images for CAM experiments are shown in the supplementary material, Figure S4. Changes in invadopodia formation rates were also verified using a standard *in vitro* invadopodia formation assay (Figure 1E and supplementary material, Figure S5).

More invasive phenotypes were also observed using transient transfections of TBX3iso1 or TBX3iso2 into T47D (luminal A; ER^+^/PR^+^/HER2^−^), SKBR3 (HER2‐enriched; ER^−^/PR^−^/HER2^+^), and MDA‐MB‐468 (basal‐like; ER^−^/PR^−^/HER2^−^) breast cancer cell lines, representing different molecular subtypes (supplementary material, Figure S6A–C). Collectively, this suggests that these findings have broad applicability and are not cell type‐specific.

The functional alterations associated with TBX3 isoform overexpression suggest a more invasive EMT phenotype. We examined the expression of several EMT markers and observed alterations in multiple proteolytic enzymes and their inhibitors (*MMP9*, *MMP14*, *PLAU* up‐regulated; *CST6* down‐regulated), as well as EMT‐associated transcription factors (*TWIST1*, *SLUG* up‐regulated) with TBX3 overexpression (Figure 1F,G). This was also observed (to a lesser extent) in shTBX3 cell lines (supplementary material, Figure S7A,B). We observed an increase in the levels of active MMP2, as assayed by gelatin zymography (supplementary material, Figure S7C), indicating functional activation of pathways involved in substrate degradation and invasiveness.

### TBX3 overexpression leads to an alteration of mesenchymal transcript levels and direct up‐regulation of SLUG

**Figure 2:**
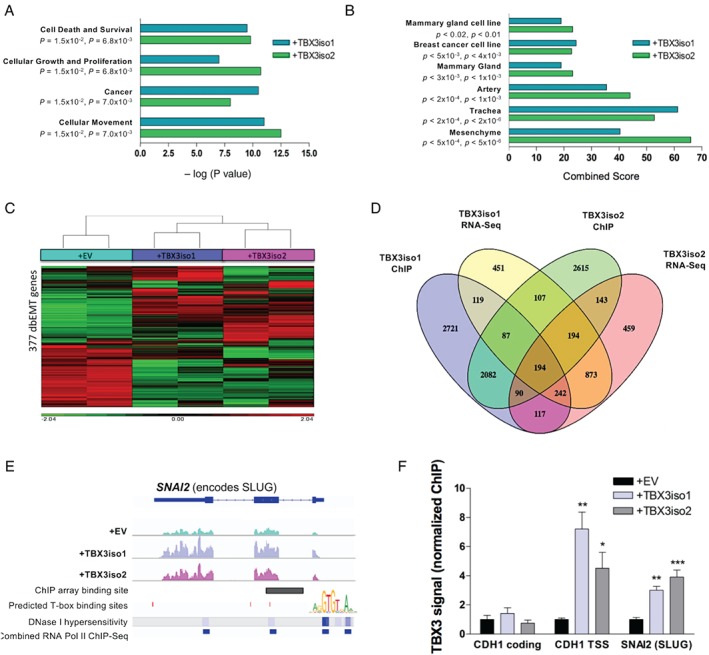
TBX3 overexpression leads to an alteration of mesenchymal transcript levels and direct up‐regulation of SLUG. RNA‐Seq of total mRNA from 21NT + EV, 21NT + TBX3iso1, and 21NT + TBX3iso2 cells in duplicate. (A) Pathway analysis was conducted using all output RNA‐Seq data for TBX3iso1‐ and TBX3iso2‐overexpressing cells and compared with the empty vector control. (B) Enrichment analysis was conducted for genes significantly altered (fold change > 1.5, FDR < 0.05). Results comparing resultant RNA‐Seq profiles with the Jensen TISSUES dataset and lowest *P*‐value tissue sites are shown. (C) Heat map of EMT genes. The dbEMT database was used to compile a list of 377 EMT genes and hierarchical clustering was conducted of RNA‐Seq data. Duplicate samples are shown for each cell line. (D) Chromatin immunoprecipitation (ChIP) experiments were conducted with TBX3 antibody or rabbit IgG. Datasets from RNA‐Seq and ChIP‐array experiments were integrated to examine the effects of TBX3 isoform binding on gene transcript levels (all fold‐change > 1.5, FDR < 0.05). (E) RNA‐Seq reads across the *SNAI2* (SLUG) gene for 21NT + EV, 21NT + TBX3iso1, and 21NT + TBX3iso2 cell lines are shown in the top three lines, followed by the identified TBX3 ChIP‐array binding site. Highly conserved T‐box binding elements (TBEs) in *SNAI2* are shown. DNAse I hypersensitivity and RNA polymerase II binding sites are shown in the bottom two lines. (F) ChIP experiments with TBX3 antibody or rabbit IgG control. Relative amounts of immunoprecipitated DNA were assessed by qPCR. E‐cadherin (*CDH1*) coding region and transcription start site (TSS) were used as negative and positive controls, respectively. The *SNAI2* primers span both the predicted overlapping TBE site identified *in silico* and the binding site from ChIP‐array experiments. Values shown represent input‐adjusted, IgG control subtracted values for the specific TBX3 IP normalized to the empty vector control. Means derived from four biological replicates were used during analysis. *p < 0.05; **p < 0.01; ***p < 0.001 by one‐way ANOVA with Tukey's *post hoc* test for comparison between three groups. Error bars represent standard deviation (SD).

To examine transcriptional changes associated with TBX3 isoform overexpression and elucidate the mechanisms involved, RNA‐Seq was conducted for TBX3iso1‐ and TBX3iso2‐overexpressing cell lines. Pathway analysis indicated that the top predicted functional changes for the TBX3 transfectants include alterations in cellular movement, cellular growth and proliferation, cell death, cell survival, and cancer‐associated processes (Figure 2A). Enrichment analysis (Enrichr) was conducted, with the input list consisting of genes significantly altered (> 1.5‐fold up or down, FDR < 0.05) for cells overexpressing either TBX3iso1 or TBX3iso2 relative to the control. Comparison to the Jensen TISSUES expression database of large‐scale tissue expression profiles revealed mesenchymal genes as most enriched (Figure 2B). Relative to the control, we detected alterations in a large proportion of EMT‐related genes compiled from the dbEMT database 28 (Figure 2C).

To determine whether the TBX3‐dependent changes in gene expression are direct, chromatin immunoprecipitation (ChIP) experiments were conducted using either TBX3 antibody or rabbit IgG antibody as a non‐specific control, and immunoprecipitated DNA was hybridized to Affymetrix Promoter 1.0R arrays. Using this approach, we identified 5652 and 5512 specific binding sites for TBX3iso1 and TBX3iso2, respectively. Comparison of TBX3 binding sites with our expression analysis identified 194 genes that were directly regulated by both TBX3 isoforms (> 1.5‐fold up or down, FDR < 0.05) (Figure 2D and supplementary material, Table S1). These 194 genes were analyzed using the PANTHER database. The lowest *P* value and corrected FDR statistic corresponded to the protease inhibitor protein class (supplementary material, Figure S8). Several other protein classes related to EMT were also altered, including extracellular matrix proteins, metalloproteases, and serine protease inhibitors. Importantly, within this list of 194 genes directly induced by TBX3 was *SNAI2* (which encodes SLUG), a transcriptional regulator and potent inducer of EMT, which may provide a mechanism for the previously described EMT and invasion‐associated functional changes in TBX3‐transfected cells.

Both TBX3 isoforms were shown to bind near an intron/exon junction of *SNAI2* based on the ChIP‐array data (Figure 2E). TBX3 has previously been shown to bind to the consensus T‐box binding element (TBE) in several different contexts 29. Consensus TBEs were investigated using the JASPAR database of transcription factor binding sites (Figure 2E and supplementary material, Figure S9A–C). A highly conserved TBE was found within the *SNAI2* gene region that coincides with binding of both TBX3 isoforms in the ChIP‐array dataset (supplementary material, Figure S7A,B). We conducted ChIP qPCR validation using primers spanning this identified TBE. Published primer sequences were used for the transcription start site (*CDH1* TSS) and coding region (*CDH1* coding) of E‐cadherin 13, representing positive and negative controls, respectively (Figure 2F). With TBX3 overexpression, there was a 3‐ to 4‐fold enrichment of TBX3 protein bound to the conserved TBE of *SNAI2*. Interestingly, this binding region overlaps with a DNase I hypersensitive region and RNA polymerase II binding region, which is suggestive of open and transcriptionally active chromatin (Figure 2E).

### SLUG up‐regulation by TBX3 is essential for increased migration and invasion

**Figure 3:**
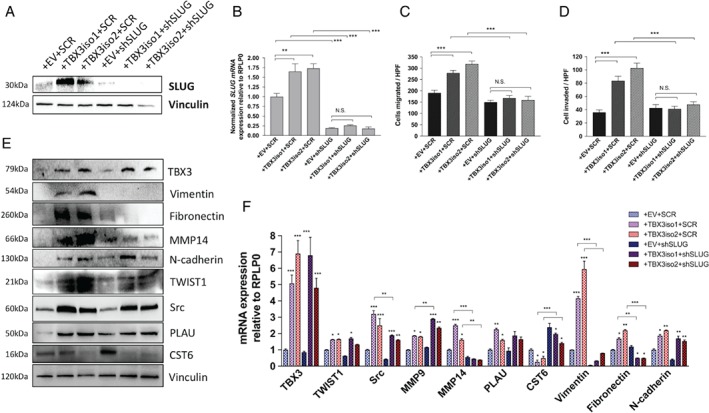
SLUG up‐regulation by TBX3 is essential for increased migration and invasion. (A) Western blot showing knockdown of SLUG protein in shRNA transductants. Protein levels were normalized to vinculin as the loading control. (B) SNAI2 transcript levels were assessed by RT‐qPCR, normalized to RPLP0 levels, and depicted as fold‐change relative to the empty vector control. (C, D) Migration and invasion assays were conducted using 8.0 μm pore Transwells coated with either gelatin (migration) or Matrigel (invasion). Cells were allowed to migrate or invade for 22 h. The number of cells that migrated or invaded per high power field is shown. Means are derived from four biological replicates. (E) Protein levels of EMT and invasion‐associated genes were assessed by western blot, normalized to vinculin. (F) mRNA levels of several EMT and invasion‐associated genes evaluated by RT‐qPCR, normalized to *RPLP0* transcript levels, and depicted as fold‐change relative to the empty vector shScramble control (+ EV + SCR). Means derived from three biological replicates were used during analysis. *p < 0.05; **p < 0.01; ***p < 0.001 by either one‐way ANOVA with Tukey's *post hoc* test or two‐way ANOVA with Tukey's *post hoc* test for comparison within and between sub‐groups, respectively. Error bars represent standard deviation.

Given the strong evidence of SLUG involvement in the induction of EMT 20, 21, we proceeded to investigate whether SLUG was essential for the phenotypes observed with TBX3 overexpression. SLUG levels were knocked down in 21NT control and TBX3 transfectant cell lines using stable shRNA‐mediated lentiviral transduction (Figure 3A,B). The scramble control (SCR) cells overexpressing either TBX3iso1 or TBX3iso2 had higher rates of migration and invasion relative to the empty vector control, similar to the levels that we previously reported in these cell lines 24. With SLUG knockdown, the rates of migration and invasion of TBX3 isoform transfectants were reduced to baseline levels, despite expressing high levels of TBX3 (Figure 3C,D).

A large proportion (7/10) of our list of EMT‐ and invasion‐associated genes previously assessed in this study were still significantly altered with high TBX3 isoform expression in cells with SLUG knockdown (Figure 3E,F). However, the induction of several key invasiveness‐associated genes up‐regulated by TBX3 was significantly impaired in the absence of SLUG, including MMP14, vimentin, and fibronectin (Figure 3E,F). Importantly, these findings suggest that there are TBX3‐induced changes in expression of EMT‐related genes that are both SLUG‐dependent and SLUG‐independent, and that the SLUG‐dependent changes are required (although not necessarily sufficient) for TBX3‐induced migration and invasion.

### TBX3 expression is elevated in low‐grade, hormone‐receptor‐positive invasive breast cancers and associated precursor lesions

**Figure 4:**
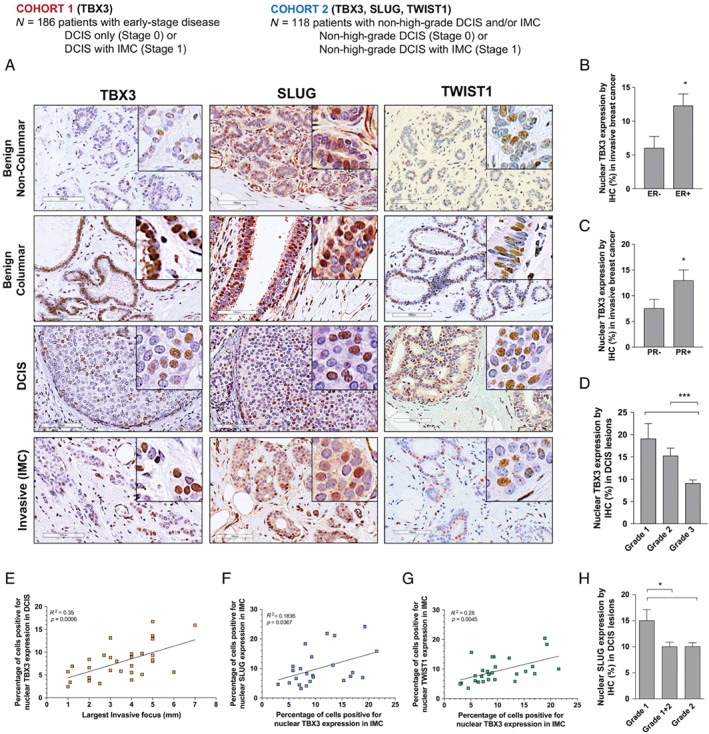
TBX3 expression is elevated in low‐grade, hormone‐receptor‐positive invasive breast cancers and associated precursor lesions. Patient samples representing cohort 1 were immunohistochemically stained and assessed for nuclear TBX3, and cohort 2 samples were stained and assessed for nuclear TBX3, SLUG, and TWIST1. (A) Representative staining patterns of cohort 2 samples (TBX3 expression and staining patterns were similar in cohort 1; not shown). (B–D) Nuclear TBX3 in cohort 1, consisting of 186 patients with early‐stage breast cancer (all nuclear grades) identified from the London Breast Cancer Database as having either DCIS only (stage 0) or DCIS with an associated early invasive component (stage I; ≤ 2 cm, and either pN0 or pN0mi). Nuclear TBX3 was compared across ductal epithelial cell types (benign non‐columnar, benign columnar, DCIS, and invasive mammary carcinoma). The association between TBX3 and (B) estrogen receptor (ER), (C) progesterone receptor (PR), and (D) DCIS nuclear grade is shown. (E–H) Nuclear TBX3, SLUG, and TWIST1 in cohort 2, consisting of 118 patients with non‐high‐grade (nuclear grade 1 or 2) DCIS, with or without associated early invasive cancer (stage 0 or stage I). Levels were compared across ductal epithelial cell types and clinical data. (E) Correlation analysis for nuclear TBX3 and size of the invasive focus in patients with stage I breast cancer. (F, G) Correlation analysis of TBX3/SLUG and TBX3/TWIST1 in the invasive carcinoma. (H) Association between nuclear SLUG and DCIS nuclear grade. *p < 0.05; **p < 0.01; ***p < 0.001 by one‐way ANOVA with Tukey's *post hoc* test for comparison between three groups, and Student's *t*‐test for comparison between two groups. Correlation and *P* values were calculated using the Pearson correlation statistic. Error bars represent SD.

From our initial characterization of effects mediated by TBX3iso1 and TBX3iso2, we observed identical EMT‐related functionality, including induction of SLUG and TWIST1 expression. We then extended our studies to human patient samples, assessing total nuclear‐localized TBX3 by immunohistochemistry (supplementary material, Figure S10) across two patient cohorts (Table 1). Our first cohort consisted of 186 pre‐invasive (stage 0, DCIS only) and early invasive breast cancer (IMC; invasive mammary carcinoma, stage I) patient samples, where we used an antibody that recognizes both TBX3 isoforms. We examined four subpopulations of cells, including benign non‐columnar, benign columnar, DCIS, and invasive cancer, after observing high expression of TBX3 in benign columnar cells (Figure 4A). Earlier studies based on genome sequencing data and mutational association suggest that columnar cell lesions (CCLs; which include columnar cell change, columnar cell hyperplasia, and flat epithelial atypia) may be an early morphologic indicator of propensity for, or a non‐obligate precursor to, the development of breast cancer 30, 31. We confirmed an association between TBX3 and estrogen receptor (ER) and progesterone receptor (PR) expression in invasive breast cancers (Figure 4B,C), which has been suggested by previous studies 32. TBX3 positivity was highest in low‐ and intermediate‐grade DCIS and significantly lower in high‐grade DCIS (Figure 4D). We then examined a second independent patient cohort of 118 patients with non‐high‐grade (low‐ and intermediate‐grade) DCIS, with or without associated early invasive cancer (stage 0 and stage I), for increased power in evaluation of TBX3 association with invasiveness of low‐grade breast cancers. In this second cohort, nuclear TBX3 expression in the DCIS was associated with the size of the invasive focus (*p* < 0.001) (Figure 4E).

We then conducted immunohistochemical staining for the EMT transcription factors SLUG and TWIST1 (which were directly and indirectly up‐regulated by TBX3, respectively) in our second patient cohort. TWIST1 was exclusively nuclear localized, and SLUG was predominantly nuclear with occasional cytoplasmic staining (Figure 4A and supplementary material, Figure S11). Each marker was assessed in all four cell populations in the same manner as for TBX3. We identified a positive correlation between TBX3 and both SLUG (*p* < 0.05) and TWIST1 (*p* < 0.001) expression in the invasive component (Figure 4F,G). Additionally, both SLUG and TWIST1 were up‐regulated in CCLs, and SLUG levels were significantly higher in low‐grade (grade 1) DCIS relative to both grade 1 + 2 (mixed) and grade 2 DCIS (Figure 4H), exhibiting staining patterns similar to TBX3. Collectively, these results suggest that TBX3 may be facilitating the process of early invasion even at the earliest stages of progression (i.e. CCLs, DCIS), and offer potential roles for downstream EMT‐related proteins such as SLUG and TWIST1 in the invasiveness of early‐stage breast cancer.

### Elevated TBX3 levels are associated with poor prognosis of breast cancer and are highly correlated with SLUG

**Figure 5:**
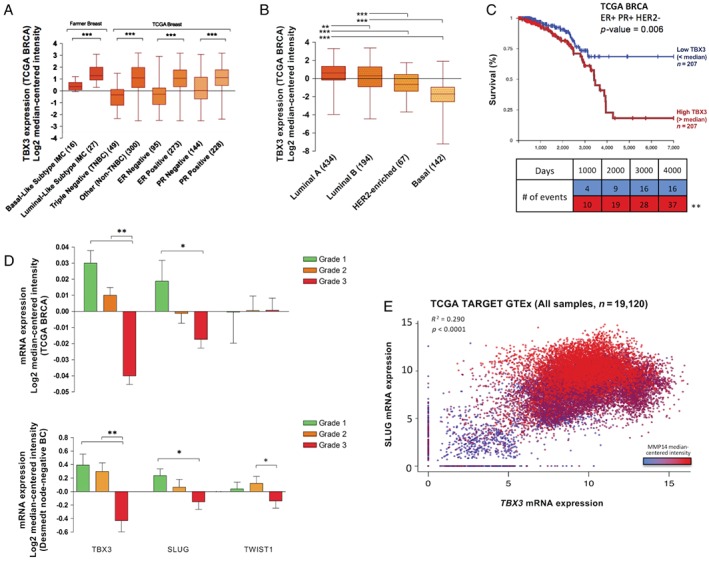
Elevated *TBX3* levels are associated with poor prognosis of breast cancer and are highly correlated with *SLUG* expression. (A, B) *TBX3* was assessed across tumor characteristics in the Farmer Breast and TCGA BRCA cohorts. *TBX3* mRNA by molecular subtype was further investigated in the TCGA BRCA dataset. (C) Kaplan–Meier survival curves for luminal A patients (ER^+^ PR^+^ HER2^−^) from the TCGA BRCA dataset, separated into high (above median) versus low (below median) *TBX3*. Number of events (breast cancer‐related deaths) is shown in the associated table. (D) *TBX3*, *SLUG*, and *TWIST1* in the TCGA BRCA and Desmedt datasets across grades. Data shown represent median‐centered, *z*‐score normalized values. (E) Correlation of *SLUG*, *TBX3*, and *MMP14* in the TCGA Target GTEx dataset consisting of 19 120 normal and tumor samples. *p < 0.05; **p < 0.01; ***p < 0.001 by one‐way ANOVA with Tukey's *post hoc* test for comparison between three groups. Survival analysis for Kaplan–Meier curve was calculated using the log‐rank test statistic. Correlation and *P* values were calculated using the Pearson correlation statistic. Error bars represent SD.

To assess the applicability of our findings in a broader breast cancer and pan‐cancer context, we compared our findings with those of available transcriptomic datasets. Using the ICGC US donor cohort, we found that *TBX3* mRNA levels were elevated in tumor cells of several cancer subtypes, including breast cancer (data not shown). By further profiling transcriptomic data from the TCGA BRCA and the Farmer Breast study 33, we identified an association of higher *TBX3* levels in luminal breast cancers, and ER‐ and PR‐positive cancers (Figure 5A,B). Survival analysis of luminal A patients in the TCGA BRCA dataset showed a statistically significant difference in survival (*p* < 0.001) between patients with high and low *TBX3* relative to the median (Figure 5C).

To examine the association between TBX3 and our identified downstream targets SLUG and TWIST1 and relate it to histopathological grade, we interrogated transcriptomics datasets with relevant grade information: TCGA BRCA (*n* = 680 with grade information) and Desmedt (*n* = 196, node‐negative breast cancers, all with grade information 34). *TBX3* and *SNAI2* mRNA levels were significantly lower in high‐grade breast cancers than in low‐ and intermediate‐grade breast cancers (Figure 5D) (*p* < 0.001), consistent with our immunohistochemical data. The Desmedt dataset also showed reduced *TWIST1* levels in high‐grade breast cancers. There was a high degree of overlap between TBX3 and SLUG in terms of patient characteristics and lesion types identified in our immunohistochemical staining and within the transcriptomics datasets. Additionally, upon examination of the TCGA TARGET GTEx dataset, we observed co‐expression of *SNAI2* and *TBX3* and tight association of *MMP14* with high levels of both transcription factors (Figure 5E). Collectively, analysis of publicly available data further supports the association and likely involvement of TBX3, along with downstream EMT transcription factors SLUG and TWIST1, in the aggressiveness of low‐grade breast cancers.

## Discussion

The use of screening mammography has drastically increased our ability to detect DCIS 35, but has been criticized as causing an overdiagnosis of breast cancer 36. Gene expression profiling has allowed successful stratification of node‐negative invasive breast cancers into low‐risk and high‐risk groups, providing information useful in clinical decision‐making for this population 37. These techniques have been extended to DCIS with some success 38, 39, 40. Additionally, panels of selected markers, including p16, COX2, and Ki67, have shown some utility in their ability to predict the behavior of DCIS in specific subsets of patients 41. Despite these efforts, there are few validated diagnostic tests or biomarkers to aid in optimization of treatment strategies for women with DCIS, and none that reliably predict risk for invasion.

We have previously established a role for TBX3 in the invasiveness of breast cancer 24. In this current study, we further explored the mechanism of this activity, particularly as associated with the phenomenon of EMT. We sought to take a high‐resolution approach to identifying downstream targets of TBX3, beginning with identification of transcriptomic changes with TBX3 overexpression and assessment of direct TBX3 binding sites. From these initial genomic studies, we identified SLUG and TWIST1 as potential downstream effectors up‐regulated with TBX3 overexpression, and SLUG as a direct downstream mediator of TBX3‐induced migration and invasion. Importantly, we have discerned expression differences in these proteins between benign non‐columnar ductal epithelial cells, CCLs, DCIS, and within the invasive carcinoma. Our examination of TBX3 by immunohistochemistry in two independent patient cohorts revealed that levels are highest in hormone receptor‐positive, low‐grade DCIS (and co‐existing CCLs) and are associated with the extent of invasion in early‐stage breast cancers. These findings, coupled with our *in vitro* data of an EMT link, are consistent with a role for TBX3 in early invasion events, likely working in conjunction with other EMT‐related factors such as SLUG and TWIST1 to facilitate a phenotype conducive to invasion.

The prevailing breast cancer progression model supported by numerous genomic and transcriptomic studies includes two divergent molecular pathways of progression – the low‐grade (ER^+^/PR^+^) and the high‐grade (ER^−^/PR^−^) pathway 23. Interestingly, sequencing and hierarchical clustering of DCIS and invasive samples have shown that samples do not cluster by diagnosis, but rather by intrinsic molecular subtype 42. This suggests that factors associated with invasiveness are distinct from histologic grade and stage and may indeed be present within the pre‐invasive DCIS 42.

**Figure 6:**
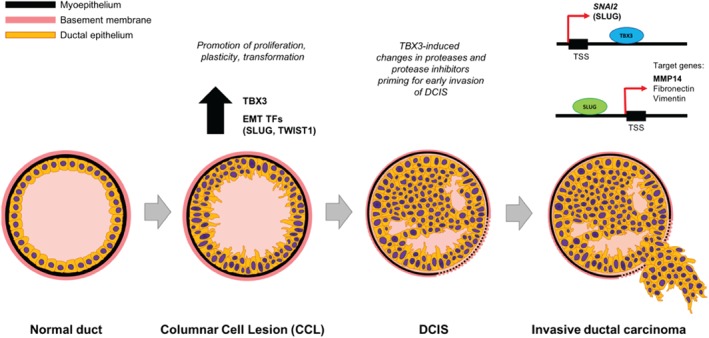
TBX3 promotes progression of pre‐invasive breast cancer cells by inducing EMT and directly up‐regulating SLUG. Proposed model through which TBX3 promotes the progression of pre‐invasive ductal carcinoma *in situ* (DCIS) lesions. TBX3 levels are low in benign non‐columnar ductal breast epithelial cells. In columnar cell lesions (CCLs), TBX3 levels are up‐regulated, with a concomitant increase in SLUG and TWIST1. This is predicted to promote proliferation and plasticity of early, pre‐invasive lesions. In a subset of low‐grade DCIS, TBX3 expression is particularly high. Overexpression of TBX3 induces altered expression of several proteases and protease inhibitors, leading to degradation of the basement membrane and priming for early invasion of DCIS into adjacent stroma. These events are facilitated by TBX3‐induced and SLUG‐dependent expression of pro‐migratory molecules such as MMP14 (membrane‐bound), fibronectin (extracellular matrix), and vimentin (intracellular).

Our proposed model of the stage‐specific role of TBX3 in early breast cancer progression is depicted in Figure 6. As observed in our immunohistochemical studies, expression of EMT‐related transcription factors (including TBX3) is low in benign non‐columnar breast epithelium. We have identified that TBX3 levels are significantly elevated in CCLs. This is in accordance with existing studies examining patterns of gene expression changes through breast cancer progression showing up‐regulation of several malignancy‐associated genes at the pre‐invasive stage 43. This pro‐EMT phenotype in CCLs, with concomitant up‐regulation of SLUG and TWIST1, is predicted to promote proliferation and plasticity of breast epithelial cells 20. There is accumulating evidence that CCLs, and in particular the CCL variant termed ‘flat epithelial atypia’ (FEA), may be non‐obligate precursors to the development of breast cancer 30, 31. Only some CCLs progress, even though TBX3 and the EMT transcription factors SLUG and TWIST1 are expressed in most (at least when observed in association with DCIS). This suggests that there are likely other changes that act in concert to allow for progression to occur.

In our present study, the significant level of enrichment of proteases, protease inhibitors, and enzyme modulators in RNA‐Seq and ChIP‐array datasets of DCIS‐like 21NT cells overexpressing TBX3 is consistent with the propensity to break down basement membrane and invade. Previous studies have suggested that the gradual loss of basement membrane, leading to changes in cell organization and polarity (rather than the acquisition of additional mutations), is the main driver in the transition from an *in situ* to an invasive phenotype 44. We have shown that induction of MMP14 with TBX3 overexpression is a SLUG‐dependent process, and this signaling cascade may be important for TBX3‐induced migration and invasion. In keeping with this, it has been previously reported that MMP14 is among the main regulators of the basement membrane transmigration process *in vivo* 45, likely due to MMP14‐dependent activation of the main type IV collagenase MMP2 46. Importantly, we have shown increased activity of MMP2 with TBX3 overexpression. Additionally, increased expression of several proteases has been shown to indirectly enhance the activity of EMT transcription factors, leading to a positive feedback loop 20, potentially resulting in high expression levels of EMT transcription factors and proteases, as we have observed both *in vitro* and in our two patient cohorts.

In conclusion, we propose a unique pathway in which TBX3 promotes progression through advancement of low‐grade DCIS to invasive carcinoma (Figure 6). Our proposed model is particularly relevant in the non‐high‐grade, ER‐positive pathway of progression. Overexpression of TBX3 at early pre‐invasive stages (CCL, DCIS) of breast cancer progression, inducing other molecular regulators of EMT (including SLUG and TWIST1), acts as an enabler to set the stage for basement membrane breakdown and invasion into adjacent stroma. Further validation of our findings in an independent cohort of stage 0 and stage I breast cancer patients and comparison with follow‐up data should be conducted in order to assess whether TBX3 expression may provide reliable risk stratification for patients diagnosed with DCIS, possibly in concert with multiple biomarkers such as Ki67, p16, COX2, and/or multi‐parameter gene expression assays 40, 41. As T‐box proteins such as TBX3 have been shown to have detrimental effects with respect to cancer progression and survival 47, a thorough understanding of the underlying mechanisms involved is crucial.

## Author contributions statement

MK, ABT, and AFC conceived the ideas, associated experiments, and contributed to interpretation of the results. MK performed the experiments and data analysis. MK and BK conducted bioinformatics analyses. MJC conducted pathological examination for immunohistochemical studies. COP conducted immunohistochemical staining for TBX3, SLUG, and TWIST1. HMH conducted a portion of the western blot experiments in Figure 3. JA conducted sequencing alignments. CDM aided in the development of methodology for *in vivo* invadopodia formation assay. KW conducted *in vitro* invadopodia formation assays. HSL provided us with CAM models. MB identified patients for immunohistochemical studies and gathered clinical information. ABT and AFC supervised the work. MK drafted the manuscript with support from ABT, BK, and JT. All the authors provided critical feedback and therefore shaped the research content and manuscript.

**SUPPLEMENTARY MATERIAL ONLINE**

**Supplementary materials and methods**

**Figure S1.** TBX3 isoform protein structure and functional domains

**Figure S2.** TBX3 expression in 21NT transfectant and 21MT‐1 transductant cell lines

**Figure S3.** Functional assessment of TBX3‐overexpressing cell lines

**Figure S4.** Representative images of cell extravasation and invadopodia formation *in vivo* in the chick chorioallantoic membrane (CAM)

**Figure S5.** TBX3‐mediated invadopodia formation

**Figure S6.** Effect of TBX3 overexpression on invasiveness in cell lines representing other breast cancer molecular subtypes

**Figure S7.** Expression of EMT markers with modulation of TBX3 levels

**Figure S8.** Protein class analysis of direct transcriptional targets of TBX3

**Figure S9.** Analysis of T‐box binding elements (TBEs) in TBX3‐bound genes identified by ChIP‐array

**Figure S10.** Subcellular localization of TBX3

**Figure S11.** H‐scores for TBX3, SLUG, and TWIST1 immunostains in various cell compartments

**Table S1.** Integrated ChIP‐array and RNA‐Seq data for assessment of direct and overlapping binding sites of TBX3iso1 and TBX3iso2

**Table S2.** Primer sequences utilized for qRT‐PCR in mRNA studies (mentioned in the supplementary material, Supplementary materials and methods)

**Table S3.** Primer sequences utilized for ChIP‐qPCR validation studies (mentioned in the supplementary material, Supplementary materials and methods)

**Table S4.** Publicly available datasets utilized for analysis (mentioned in the supplementary material, Supplementary materials and methods)

## Supporting information


**Supplementary materials and methods**

**Figure S1.** TBX3 isoform protein structure and functional domains.
**Figure S2.** TBX3 expression in 21NT transfectant and 21MT‐1 transductant cell lines.
**Figure S3.** Functional assessment of TBX3 overexpressing cell lines.
**Figure S4.** Representative images of cell extravasation and invadopodia formation *in vivo* in the chick chorioallantoic membrane (CAM).
**Figure S5.** TBX3‐mediated invadopodia formation.
**Figure S6.** Effect of TBX3 overexpression on invasiveness in cell lines representing other breast cancer molecular subtypes.
**Figure S7.** Expression of EMT markers with modulation of TBX3 levels.
**Figure S8.** Protein class analysis of direct transcriptional targets of TBX3.
**Figure S9.** Analysis of T‐box binding elements (TBEs) in TBX3‐bound genes identified by ChIP‐array.
**Figure S10.** Subcellular localization of TBX3.
**Figure S11.** H‐scores for TBX3, SLUG, and TWIST1 immunostains in various cell compartments.

**Table S1.** Integrated ChIP‐array and RNA‐Seq data for assessment of direct and overlapping binding sites of TBX3iso1 and TBX3iso2

**Table S2.** Primer sequences utilized for RT‐qPCR in mRNA studies (mentioned in the supplementary material, Supplementary materials and methods)
**Table S3.** Primer sequences utilized for ChIP‐qPCR validation studies (mentioned in the supplementary material, Supplementary materials and methods)
**Table S4.** Publicly available datasets utilized for analysis (mentioned in the supplementary material, Supplementary materials and methods)
