# Supplementary material for: TBX3 promotes progression of pre‐invasive breast cancer cells by inducing EMT and directly up‐regulating SLUG
Source: J Pathol. 2019 Apr 8;248(2):191–203. doi: 10.1002/path.5245 (PMC6593675; doi:10.1002/path.5245)
Supplement: Supplementary file 1 — Supplementary materials and methods [file PATH-248-191-s001.docx]

**TBX3 promotes progression of pre-invasive breast cancer cells by inducing EMT and directly up-regulating SLUG**

Krstic M *et al*. *J Pathol* DOI: 10.1002/path.5245

**Supplementary materials and methods**

Reference numbers refer to the main text reference list

# *Stable transfection*

21NT parental cell lines were seeded into six-well plates at 350 000 cells per well and transfected the following day at 3 μg of DNA plasmid per well using the Lipofectamine 3000 Transfection Kit (L3000; Invitrogen, Carlsbad, CA, USA). Plasmid DNA constructs have been previously described [24] and consist of an empty vector (EV), TBX3iso1, and TBX3iso2 construct within a pcDNA3.1 (V79020; Invitrogen) vector. Selection was performed using αHE medium supplemented with 500 μg/ml G418. Resistant clones were pooled, expanded, and frozen for later use.

# *Transient transfection*

For assessment of E-cadherin expression and localization, 21NT parental cell lines were seeded into six-well plates at 350 000 cells per well and transfected the following day with 3 μg of DNA plasmid per well using the Lipofectamine 3000 Transfection Kit (L3000; Invitrogen). Plasmid DNA constructs consisted of an empty vector (EV), TBX3iso1, and TBX3iso2 construct within pZsGreen1-N1 vector containing the ZsGreen reporter gene. For immunofluorescence studies, transfected cells were grown under normal conditions for 72 h and then underwent immunofluorescent staining.

For invasion assay studies, cells (T-47D, SKBR3, MDA-MB-468) were incubated with transfection reagent and the appropriate plasmid for 24 h, then harvested, and assays were performed. Plasmid DNA constructs consisted of an empty vector (EV), TBX3iso1, and TBX3iso2 construct within pZsGreen1-N1 vector containing the ZsGreen reporter gene.

# *Lentiviral transduction*

The generation of shRNA-encoding lentivirus particles for TBX3 knockdown was conducted as previously described [24]. The shRNA target sequence for TBX3 was GCATACCAGAATGATAAGATA, which targeted the coding sequence of both TBX3iso1 and TBX3iso2. The shRNA target sequence for Luciferase (representing an off-target control) was ACGCTGAGTACTTCGAAATGT. The lentivirus particles were used to generate 21MT-1 shLUC and 21MT-1 shTBX3 cell lines. Selection was performed using αHE medium supplemented with 0.8 μg/ml puromycin. Resistant clones were pooled, expanded, and frozen for later use.

Down-regulation of SLUG (*SNAI2*) was performed using lentivirus-mediated shRNA transduction. 293T packaging cells were seeded at 80% confluence in six-well plates with low-antibiotic growth media (DMEM with 10% FBS and 0.1× penicillin/streptomycin). The following day, the packaging cells were transfected with the packaging plasmid (pCMV-dR8.91, 900 ng; Addgene, Watertown, MA, USA), envelope plasmid (VSV-G/pDM2.G, 100 ng; Addgene), and psi-LVRU6H vector encoding one of four shRNAs specific to *SNAI2* (HSH017502-LVRU6H, 1 μg; Genecopoeia, Rockville, MD, USA) or an empty vector control (CSHCTR001-LVRU6H, 1 μg; Genecopoeia) (quantities reported per well). The FuGENE transfection reagent (E2311; Promega, Madison, WI, USA) was mixed with the three-plasmid mix as per the manufacturer’s protocol. The transfection mix was incubated for 30 min at room temperature, and 200 μl was added to the packaging cells. Viral harvesting was done three times, spaced out by 12–24 h by replacing media with 6 ml of high-BSA growth media (20 g of BSA per 100 ml of DMEM). The three viral harvests were pooled and the viral supernatant was frozen.

21NT transfectants (21NT + EV, 21NT + TBX3iso1, 21NT + TBX3iso2) were seeded at 200 000 cells per well in six-well plates. The following day, 300 μl of the shRNA-encoding lentiviral particles was added to the cells along with 2 μg of polybrene (TR-1003-G; Sigma, St Louis, MO, USA) per 2 ml of media. The cells were incubated at 37°C overnight and transduced cells were selected for by including 500 μm hygromycin (10687-010; Invitrogen) in the media (aHE10F with 500 μm hygromycin, 500 μg/ml G418). Media were replaced again the following day, and cells were trypsinized and expanded into T25 flasks on the third day.

# *Preparation of 21T protein lysates*

Protein lysates were prepared by adding 1 ml of RIPA buffer (150 mm NaCl, 1% Triton-X, 0.5% deoxycholic acid, 0.1% SDS, 50 mm Tris-base; pH 8.0) with Halt Protease Inhibitor Cocktail (78429; Thermo Scientific, Waltham, MA, USA) to confluent 10 cm plates. Cells were scraped, collected in a microcentrifuge tube, and incubated on ice for 10 min. Lysates were then centrifuged at 13 000 rpm for 10 min at 4°C. The supernatant was collected and protein concentrations were determined using a Bradford Protein Assay kit (5000002; BioRad, Hercules, CA, USA) by comparing to BSA standards.

# *Subcellular fractionation*

Cells (1.5 × 10^6^) were seeded onto 10 cm plates. The following day, 500 μl of subcellular fractionation buffer [250 mm sucrose, 20 mm HEPES (pH 7.4), 10 mm KCl, 1.5 mm MgCl_2_, 1 mm EDTA, 1 mm EGTA, 1 mm DTT, and 1× protease inhibitors (78429; Thermo Scientific)] was added to the cells. The cells were scraped immediately, transferred to a microcentrifuge tube, passed through a 25-gauge needle ten times, and left on ice for 20 min. The cell lysate was centrifuged at 3000 rpm for 5 min, resulting in (1) a nuclear pellet, and (2) a supernatant consisting of cytosolic and membrane fractions. For the nuclear fraction, the pellet was washed once with 500 μl of subcellular fractionation buffer, passed through a 25-gauge needle ten times, and centrifuged again at 3000 rpm for 10 min to produce a pure nuclear fraction. The nuclei were then resuspended in nuclear lysis buffer [50 mm Tris HCl (pH 8.0), 10 mm EDTA, 0.1% SDS, and 10% glycerol], sonicated for 90 s (continuous), and passed through a 30 G needle five times. For the cytosolic fraction, the supernatant was centrifuged at 8000 rpm and the remaining supernatant was saved.

# *Preparation of conditioned media and western blotting*

For assessment of secreted protein expression (MMP9), conditioned media were prepared by seeding 1.0 × 10^6^ cells into T75 flasks, and cells were maintained in low serum media (αMEM with 0.1% FBS) for 48 h. Media were collected and concentrated 25× using centrifugal filters (Amicon Ultra-4, UFC803024). Protein was resolved (40 μg) on a 10% SDS-PAGE gel. Immediately after transferring proteins to a PVDF membrane, total protein was visualized by Ponceau Stain using the BioRad ChemiDoc imaging system, and an image of the membrane was acquired for subsequent normalization. The membrane was then de-stained and MMP9 protein levels were assessed by western blot (specifications can be found in the ‘Electrophoresis and western blotting’ section).

# *Electrophoresis and western blotting*

Protein (10–50 µg) was resolved on a 10% SDS-PAGE gel. The membrane was blocked with 5% milk for 1 h (or 5% BSA where specified), then incubated with primary antibody overnight at 4°C: anti-TBX3 (Invitrogen; 424800; 1:1000), anti-SLUG (Abcam; ab27568; 1:1000 in BSA), anti-vimentin (Abcam; ab92547; 1:1000), anti- fibronectin (Abcam; ab2413; 1:1000), anti-uPa (Abcam; ab133563; 1:250), anti-MMP9 (Abcam; ab38898, 1:1000), anti-MMP14 (Abcam; ab51074; 1:500), anti-Src (Cell Signaling, Danvers, MA, USA; 2110; 1:1000), anti-pSrc (Y416) (Cell Signaling; 2101; 1:1000 in BSA), anti-pSrc (Y527) (Cell Signaling; 2105; 1:1000 in BSA), anti-TWIST1 (Abcam; ab50887; 1:100 in BSA), anti-CST6 (Sigma; WH0001474M1; 1:1000), anti-N-cadherin (Abcam; 18203, 1:1000), anti-vinculin (Sigma; V9264; 1:20 000), and anti-H4 (EDM Millipore; 05-858, 1:1000). The membrane was incubated with secondary antibody conjugated to HRP, either anti-rabbit (Sigma, A0545; 1:1000) or anti-mouse (GE Healthcare, NXA931; 1:1000) as appropriate, for 1 h at room temperature. The western blots were visualized using Luminata Crescendo (EDM Millipore, WBLUR0500) and images were taken using the BioRad ChemiDoc imaging system. Densitometric quantification was performed using ImageLab (BioRad), and quantities were normalized to vinculin expression. Where quantifications of western blots are shown, data were acquired through densitometric quantifications across three biological replicates.

# *Immunofluorescence*

ZsGreen vector transiently transfected cells (described above) were fixed with 10% neutral buffered formalin for 10 min, and permeabilized with 0.5% Triton X-100 in PBS for 10 min. Blocking was conducted with normal goat serum (Invitrogen, 50-0627) at room temperature for 1 h. Rabbit anti-E-cadherin antibody (BD Biosciences, San José, CA, USA; 610181) was added at a dilution of 1:150 in 10% normal goat serum and was incubated overnight at 4°C. The following day, cells were washed three times with PBS, and secondary antibody Alexa647 Goat Anti-Rabbit IgG (Invitrogen, A21245) was added at a dilution of 1:1000 in 10% normal goat serum and incubated for 1 h at room temperature. Cells were washed five times with PBS. Hoechst 33342 (Invitrogen, H1399) diluted to 1:5000 in PBS was incubated for 30 min at room temperature. Cells were washed once with PBS. Coverslips were mounted with ProLong Gold Antifade Reagent (Invitrogen, P36930). Images were captured at 40× magnification using an Olympus Confocal Imaging System (FluoView FV1000 coupled to the IX81 Motorized Inverted System Microscope). Images were converted to 8-bit images by separating channels. Mean grey area was quantified within the red channel across ZsGreen-positive (transfected) cells. E-cadherin expression across the cell lines was normalized to the empty vector control.

# *Scratch wound (migration) assay*

Cells were cultured to near-confluence in six-well plates and starved in low serum media (αMEM with 0.1% FBS) for 16 h. A 2-mm-wide scratch was then made in the confluent monolayer. The cells were rinsed with PBS and fresh media (αMEM media with 10% FBS) were added. Cell migration was visualized and phase contrast images were taken at 10× using the Olympus IX70 inverted microscope after 6, 12, and 24 h. Cell migration was calculated by comparing the wound area at the specified time points using ImageJ (Open source, https://imagej.nih.gov/ij/). Means derived from four biological replicates were used during analysis.

# *Transwell invasion assay*

Transwell invasion assays were conducted to examine the invasive potential of the transfected cell lines. Stably transfected 21NT cells (representing luminal B subtype) were used for initial studies. For stable 21NT transfectant cells, 50 000 cells per 100 μl of media were added to the upper chamber of an 8.0 μm pore Transwell insert (Corning, 3422; 24-well plate) coated with 10 μg of Matrigel (Corning, 354480). After incubation at 37°C and 5% CO_2_ for 18 h, the membranes were fixed with 1% glutaraldehyde for 20 min and stained with full-strength hematoxylin for 15 min. Cells that did not migrate and remained on the upper surface of the membrane were removed using a cotton swab. Images of five non-overlapping fields of view were acquired using an Olympus IX70 inverted microscope at 10× objective. Cells were counted from the acquired images using ImageJ. Means derived from four replicates were used during analysis.

Invasiveness of transiently transfected cells was also assessed, including T-47D (luminal A), SKBR3 (HER2-enriched), and MDA-MB-468 (basal-like) cells transfected with either an empty vector (EV), TBX3iso1, or TBX3iso2 construct within the pZsGreen1-N1 vector containing a ZsGreen reporter gene. Twenty-four hours post-transfection, cells were trypsinized, harvested, and suspended in their respective media in the absence of FBS and addition of 0.1% BSA. Cells were then added to the upper layer of an 8.0 μm Transwell insert coated with 10 μg of Matrigel at 50 000 cells per 100 μl of media. After incubation at 37°C and 5% CO_2_ for 18 h, the membranes were fixed with 1% glutaraldehyde for 20 min. Cells that did not migrate and remained on the upper surface of the membrane were removed using a cotton swab. Green fluorescent images and bright-field images of three non-overlapping fields of view were acquired and superimposed using an EVOS FL Auto microscope at 10× objective. The number of green cells was determined using ImageJ software. Means derived from four biological replicates were used during analysis.

# *Cell–cell adhesion assay*

The cell–cell adhesion assay was carried out as described previously [13]. Briefly, T75 plates were rinsed twice with PBS and cells were dissociated using 3 mm EDTA. Cells were resuspended in αHE10F media and passed through a cell strainer to dissociate cell clusters. Cells (200 000) were added to the appropriate media (αHE10F with 3 mm EDTA or αHE10F with 1 mm CaCl_2_) in a 6 cm Petri dish. Plates were incubated at 37°C on a shaking platform for 30 min. After this incubation period, ten non-overlapping fields of view per dish were captured using the Olympus IX70 inverted microscope at 10× objective. Clusters containing more than four cells were counted, and counts from ten fields of view were added together for each plate. Means derived from four biological replicates were used during analysis.

# *Gelatin zymography*

Cells (1.5 × 10^6^) were seeded onto 10 cm plates. The following day, media were replaced by 10 ml of serum-free αHE media with 500 µg/ml G418, and cells were serum-starved for 24 h. Media were collected and concentrated 25× using centrifugal filters (Amicon Ultra-4, UFC803024). Equal volumes (normalized to 1.5 × 10^6^ cells) of the conditioned media were loaded into a 10% Criterion Zymogram gel (BioRad, 3450080) without boiling/reducing and resolved at 4°C. To remove SDS from the gel, the gel was washed with renaturation buffer (2.5% Triton X-100) twice for 30 min each, followed by two 20-min washes with distilled water. The gel was then incubated in development buffer [50 mm Tris, 200 mm NaCl, 5 mm CaCl_2_ (anhydrous), 0.02% Brij-35, pH 7.5] at 37°C for 16 h. The gel was then stained (40% methanol, 10% acetic acid, 0.5% Coomassie Blue R-250) for 1 h at room temperature with gentle agitation, followed by de-staining (40% methanol, 10% acetic acid) until clear bands appeared. Images were taken using the BioRad ChemiDoc imaging system. Reverse images were used for quantification. Means derived from three biological replicates were used during analysis.

# *Chick chorioallantoic membrane extravasation assays*

Cells were pre-labeled with 1 µm CellTracker conjugated to Green CMFDA Dye (Thermo Fischer Scientific, C7025) and washed once with PBS. Using the chorioallantoic membrane (CAM) of the chicken embryo, embryonic day 12 embryos were intravenously (i.v.) injected with 50 000 cells (50 µl of 1×10^6^ cells per ml cell suspension).

For the extravasation assays, rhodamine-labeled lectin (75 µl of 0.1 mg/ml, RLK-2200; Vector Laboratories Inc, Burlingame, CA, USA) was i.v. injected 10 min after injection of fluorescently labeled cells in order to visualize the luminal surface of endothelial cells in the CAM. Immediately following i.v. injection of lectin-rhodamine, a 1 cm × 1 cm field of the CAM marked by foil markers at the corners was made for each animal and the number of cells that were intravascular, in the process of extravasation and extravasated into the CAM stroma, was counted at *T* = 10 min and *T* = 24 h. A resonance scanner confocal microscope (Olympus FluoView FV1000 coupled to the IX81 Motorized Inverted System Microscope) was used to determine each cell’s location within the CAM with respect to lectin-rhodamine signal in real time. All quantifications were conducted blinded. Values reported represent the percentage of cells that successfully extravasated in the 1 cm × 1 cm field across 30 biological replicates. All quantifications were conducted blinded.

For *in vivo* invadopodia assays, the chick embryos were returned to their incubators after injection of labeled cells, and at the 6-h time-point were i.v. injected with rhodamine-labeled lectin (75 µl of 0.1 mg/ml; Vector Laboratories Inc) in order to visualize the luminal surface of the endothelial cells in the CAM. A resonance scanner confocal microscope was used to determine each cell’s location within the CAM with respect to lectin-rhodamine signal in real time. By scanning across the *Z*-axis, green fluorescently labeled cells were assessed for the presence of functional invadopodia protruding between the intravascular/extravascular boundary. Values reported represent the percentage of cells displaying functional invadopodia *in vivo* across six biological replicates consisting of approximately 30 scanned cells each. All quantifications were conducted blinded.

# *Invadopodia formation assay*

The *in vitro* invadopodia formation assay was conducted as previously described [25]. Briefly, coverslips were coated with 1 ml of cold poly-l-lysine (Sigma, P7405; 50µg/ml in PBS) and aspirated after 20 min, followed by a 15-min incubation with 0.5% glutaraldehyde. Coverslips were then inverted on an 80 µl drop of 0.2% gelatin in PBS labeled with Alexa594 (Thermo Fischer Scientific, G13187) and incubated for 10 min. Coverslips were lifted, then incubated with 5 mg/ml sodium borohydride for 15 min to quench reactive groups in the gelatin matrix, followed by extensive washing with PBS. Cells were trypsinized and plated on coverslips for 12–24 h (as optimized), followed by fixation with 4% paraformaldehyde and permeabilization with 0.1% Triton X-100 in PBS. Samples were blocked with 5% skim milk powder, followed by staining with Alexa488-labeled phalloidin (Thermo Fischer Scientific, A12379) to label F-actin. Cells were imaged using the Nikon Fast A1R Upright Microscope. Cells with actin cores overlying spots of degradation were counted as invadopodia-forming cells. The percentage of cells forming invadopodia was quantified from four biological replicates, assessing invadopodia formation in 10–20 random, non-overlapping fields.

# *RNA isolation and quantitative real-time PCR (qRT-PCR)*

RNA was isolated using the RNeasy Mini Kit (Qiagen, 74104). RNA (500 ng) was transcribed into cDNA using the qScript cDNA SuperMix (Quanta Biosciences, 84034). The RT^2^ SYBR Green ROX qPCR Mastermix (Qiagen, 330521) was utilized for quantitative PCR with the primer sequences as listed in Table S2. The output Ct values were normalized to GAPDH and RPLP0 expression for 21NT transfected and transduced cells, respectively, using the ∆∆Ct method. Means derived from a minimum of three biological replicates were used during analysis.

# *Chromatin immunoprecipitation*

Twenty million cells (per replicate) were grown to confluence for each cell line. The cells were washed twice with cold PBS and then cross-linked with 10 ml of PBS with 1% formaldehyde solution for 15 min at room temperature on a rocking platform. The reaction was quenched with 1 ml of 2.625 m glycine. The cells were then scraped into the supernatant and washed twice with cold PBS.

Pellets were resuspended in 1 ml of nuclei isolation buffer [50 mm Tris (pH 8.0), 60 mm KCl, 0.5% NP-40] with protease inhibitors (Thermo Scientific, 78429) and incubated on ice for 10 min, then spun at 3000 rpm for 3 min at 4°C for isolation of nuclei. Nuclei were resuspended in 1 ml of Lysis Buffer [0.5% SDS, 10 mm EDTA, 0.5 mm EGTA, 50 mm Tris HCl (pH 8.0)] with protease inhibitors. Chromatin was sonicated for 40 min at 2°C (using Diagenode Bioruptor Pico; 30 s on, 30 s off). The sonicated samples were centrifuged at 13 000 rpm for 10 min at 4°C to remove cell debris. Sonicated chromatin was then pre-cleared with 200 μl of Protein-A Dynabeads (Life Technologies, Carlsbad, CA, USA; 10002D) for 1 h at 4°C on a rocking platform. Simultaneously, 50 μl of Protein-A Dynabeads was washed with 0.5% BSA–PBS and incubated with 5 μg of normal rabbit IgG antibody (Invitrogen, 10500C) or 5 μg of rabbit anti-TBX3 antibody (Invitrogen, 424800) in 100 μl of dilution buffer (PBS with 0.2% Tween 20 and 1% BSA) for 2 h at room temperature on a rocking platform (quantities listed per biological replicate). The bead–antibody complex was then washed twice with 0.5% BSA–PBS, resuspended in dilution buffer, and then added to the sample. Immunoprecipitation was conducted overnight (16 h) at 4°C on a rocking platform.

Bound chromatin was eluted the following day. Each wash step was performed at 4°C for 5 min on a rocking platform. Chromatin was washed with Wash Buffer I (20 mm Tris HCl, 150 mm NaCl, 2 mm EDTA, 1% Triton X-100, 0.1% SDS) twice, Wash Buffer II (20 mm Tris HCl, 500 mm NaCl, 2 mm EDTA, 1% Triton X-100, 0.1% SDS) once, and TE buffer (10 mm Tris HCl, 1 mm EDTA, pH 8.0) twice. Bound chromatin was eluted using 200 μl of Elution Buffer (100 mm NaHCO_3_, 1% SDS) at 65°C for 20 min. NaCl was added to a final concentration of 200 mm and incubated with the chromatin overnight at 65°C for de-crosslinking. Proteinase K (Thermo Scientific, AM2546) was added and incubated for 2 h at 50°C, followed by RNase A (Thermo Scientific, 12091039) treatment for 60 min at 37°C for protein and RNA degradation, respectively. The DNA was purified using the QIAquick PCR Purification Kit (Qiagen, 28106) and eluted with 20 μl of elution buffer.

For ChIP array purposes, the eluted DNA was amplified using the Whole Genome Amplification Kit (Sigma, WGA2) as per the manufacturer’s protocol, with the omission of the fragmentation step. In order to obtain sufficient DNA for ChIP-array hybridization, the amplified DNA was re-amplified using the GenomePlex Complete Whole Genome Amplification kit (Sigma, WGA2) with the incorporation of dUTP (Jena Bioscience, Jena, Germany; NU-1021S) at a final concentration of 2 mm, and dTTP was reduced to 8 mm. The DNA was once again purified using the QIAquick PCR Purification Kit.

For validation of select ChIP targets, 5 μl of the eluate was used for qPCR. The % input method was utilized for quantification of enrichment. An input of 5% was saved prior to immunoprecipitation and was de-crosslinked and purified as described above. The Ct values for the input were subtracted from the Ct values of the immunoprecipitated samples. A fold change was then calculated consisting of TBX3 IP over IgG control (signal over noise) to allow for easy comparison between primer sets. The primers used for ChIP-qPCR experiments are listed in Table S3. The CDH1 transcription start site (TSS) and coding region served as the positive and negative control, respectively. The CDH1 control primer sequences were obtained from a previously published study reporting the TBX3 binding site within E-cadherin in melanoma cell lines [13].

# *RNA-Seq*

Cells were grown to 80% confluence and RNA was isolated using the RNeasy Mini Kit (Qiagen, 74104) incorporating DNase I digestion (Qiagen, 79254). Samples were sent to the Donnelly Sequencing Centre (Toronto, Canada) and assessed for RNA integrity using an Agilent bioanalyzer. For each sample, stranded mRNA libraries were prepared and sequenced on an Illumina HiSeq 2500 sequencer using V4 chemistry. Paired-end read length was 51 base pairs. Raw reads were then uploaded to Basespace (https://basespace.illumina.com/) and imported into Galaxy software (https://usegalaxy.org). The FastqGroomer tool within Galaxy was used to prepare the files for alignment, and the TopHat tool within Galaxy was used to align the FASTQ files to the reference genome (Hg19) using the default parameters, except that the maximum number of alignments was capped at 10. Aligned sequences were imported as .bam files into Partek Genomics Suite (PGS; <http://www.partek.com/pgs)> software and analyzed using the RNA-Seq analysis workflow. Gene reads were normalized for transcript length using the RPKM normalization algorithm*,* and differentially expressed genes between TBX3iso1 and TBX3iso2 compared with empty vector were determined using ANOVA statistical tests. For each comparison, final gene lists were created using a minimum fold change cut-off of 1.5-fold, and a maximum false discovery rate (FDR) of 0.05 (step-up FDR algorithm of Benjamini and Hochberg).

# *ChIP promoter arrays*

Chromatin was immunoprecipitated as described above. The amplified product (7.5 μg) was fragmented, labeled, and hybridized to the GeneChip Human Promoter 1.0R Array (Affymetrix, 900776), and the hybridized array was scanned as per the manufacturer’s instructions. All microarray work was carried out at the London Regional Genomics Centre (Robarts Research Institute, London, Ontario, Canada). Resultant .cel files were then imported into Partek Genomic Suites (PGS) and analyzed using the Chromatin Immunoprecipitation workflow. Differential probe intensities between TBX3iso1 and TBX3iso2 versus empty vector were determined by ANOVA and enriched regions of significance were determined using the MAT algorithm. To be further considered, an enriched region had to have a minimum MAT score of 5 at *p* < 0.05.

# *Bioinformatics analysis*

To compare RNA-Seq and ChIP-array datasets, gene lists were constructed using Venny 2.1 (Spanish National Biotechnology Centre; [http://bioinfogp.cnb.csic.es/tools/venny/).](http://bioinfogp.cnb.csic.es/tools/venny/)) Genes of interest include those significantly altered with overexpression of both TBX3 isoforms by RNA-Seq (fold change > 1.5 relative to the empty vector, *p* < 0.05) and genes which both TBX3 isoforms directly bound in ChIP-array datasets (MAT score > 5, *p* < 0.05). Altered biological functions with TBX3 isoform overexpression were examined using Ingenuity Pathway Analysis (IPA) (Qiagen) by examining RNA-Seq data. Gene reads and ChIP binding locations were visualized using Interactive Gene Viewer (IGV) software (Broad Institute). Consensus binding motifs of highly conserved T-box proteins (TBX1, TBX2, TBX4, TBX5, TBX15, TBX20, TBX21) within the JASPAR database (University of Copenhagen Centre for Molecular Medicine and Therapeutics; http://jaspar.genereg.net) were assessed, and coordinates that had overlap for all aforementioned related T-box transcription factors were imported into IGV. DNase I hypersensitivity and RNA polymerase II ChIP tracks were also imported into IGV. A cut-off threshold of more than 40 for each coordinate occurring in the RNA pol II (POLR2A) ChIA-PET combined dataset in various cell lines was introduced to assess consistent binding sites. The aforementioned ENCODE tracks are listed in Table S4.

For conservation analysis, the T-box binding motif (GAGGTGTCAGA) was identified within the *SNAI2* gene (chr8:49,832,796-49,832,806 – hg19). Using the NCBI Basic Local Alignment Search Tool (BLAST), this sequence, along with 10 bp upstream and downstream of the motif (GTCCTTGGAGGAGGTGTCAGATGGAGGAGG), was analyzed using a list of available UCSC species. A subset of matches was manually curated and aligned with differences highlighted. For T-box motif analysis, the DNA sequence of regions bound by both isoforms was determined by extracting sequence programmatically from UCSC (hg19), where ‘CHR’, ‘START’, and ‘END’ indicate location of the region of interest. A search for the TBX3 motif was performed in obtained sequences and genes containing motif matches were highlighted.

The TCGA BRCA, Farmer Breast, and Desmedt Breast datasets were interrogated and data were exported using XenaBrowser (University of California, Santa Cruz; https://xenabrowser.net/datapages/). The aforementioned datasets are listed in Table S4.

The PANTHER database (University of Southern California; http://pantherdb.org) was used to conduct over-representation analysis using Fischer’s exact test with FDR multiple test correction and focusing on protein class and Gene Ontology (GO) biological pathways.

Enrichr pathway analysis (Icahn School of Medicine, Mount Sinai; <http://amp.pharm.mssm.edu/Enrichr/)> was conducted with the input list consisting of 194 genes significantly altered (fold change > 1.5, *p* < 0.05) for cells overexpressing either TBX3iso1 or TBX3iso2 relative to the empty vector control from the normalized RNA-Seq data. Results assessing Jensen TISSUES expression data are reported. The combined score takes into account the *P* value and *z* value, with the calculation CombinedScore = ln(*p*)**z*, with *p* representing the *P* value and *z* representing the *z*-score [26].

# *Immunostaining of cell pellets*

Cell pellets were prepared and immunostained as previously described [24]. Briefly, cells were trypsinized, washed twice in PBS, and resuspended in 10% neutral buffered formalin (NBF) and stored at 4°C overnight. The next day, NBF was removed, and pellets were resuspended in 1 ml of 1% agarose. The hardened pellets were wrapped in lens paper, casetted, and processed to paraffin. Sections 4 μm thick were prepared and immunostained with rabbit anti-TBX3 antibody (Abcam, ab99302) diluted 1:300 at 4°C overnight. Signal was developed using DAB and slides were counterstained in Harris’s hematoxylin.

# *Immunohistochemistry*

Formalin-fixed, paraffin-embedded tissues were sectioned at 4.0 µm and added to a charged glass slide. Sections were deparaffinized, rehydrated, and incubated in TBS-Tween or TBS-Triton [Tris buffered saline (TBS) + 0.5% Tween-20 for TBX3 and SLUG stains; TBS + 0.1% Triton-X for TWIST1 stain] for 20 min for membrane permeabilization. Heat-induced antigen retrieval was conducted with citrate buffer (pH 6.0) for 20 min. The UltraVision LP Detection System (Thermo Fisher Scientific, TL-015-HD) was used with optimization of incubation length. Endogenous peroxidase activity was blocked for 10 min for all samples. For the TBX3 stain, slides were blocked for 5 min and incubated in TBX3 primary antibody (Abcam, ab99302; recognizes both TBX3 isoforms) at 1/200 dilution for 15 min at room temperature, and 11 min in the HRP polymer. For the SLUG stain, slides were blocked for 6 min using the UltraVision protein block and an additional 30 min using the Dako protein block (Dako, CD310081) in order to reduce non-specific background staining. Slides were incubated in SLUG primary antibody (Abcam, ab27568) at 1/750 dilution for 70 min at room temperature and 15 min in the HRP polymer. For the TWIST1 stain, slides were blocked for 6 min and incubated in TWIST1 primary antibody (Abcam, ab50887) at 1/200 dilution for 1 h at room temperature, 10 min in primary antibody enhancer, and 15 min in the HRP polymer. Signal for all markers was developed using DAB and slides were counterstained in Harris’s hematoxylin.

# *Quantification of immunohistochemistry*

Slides were stained and scanned using an Aperio AT2 slide scanner (Leica Biosystems). Full scanned digital slides were analyzed by an anatomical pathologist, blinded to the diagnosis, and representative images were captured at 20× magnification using the Aperio ImageScope slide viewing software. Each image represented one of four tissue compartments including benign non-columnar cells, benign columnar cell lesions (CCLs; which include columnar cell change, columnar cell hyperplasia or flat epithelial atypia), ductal carcinoma *in situ* (DCIS), and invasive cancer. The expression of each molecular marker was assessed using the ImmunoRatio plugin (University of Tampere, Finland) for ImageJ to assess the percentage of positive cells. For each image, the cell type of interest was solely analyzed and the remaining cells (i.e. stromal cells) were cropped out. A control slide representing serial sections was run for each batch of slides (with quantification and statistical analysis) to ensure identical and reproducible staining for each run.

# *Statistical analysis*

Statistical analyses were conducted using GraphPad Prism 5. One-way ANOVAs were used for experiments containing three or more groups, with either Tukey’s or Dunnett’s *post hoc* test. *t*-tests were used for experiments containing two groups. Error bars represent standard deviation. *P* values less than 0.05 were considered statistically significant.
