# Supplementary material for: TBX3 promotes progression of pre‐invasive breast cancer cells by inducing EMT and directly up‐regulating SLUG
Source: J Pathol. 2019 Apr 8;248(2):191–203. doi: 10.1002/path.5245 (PMC6593675; doi:10.1002/path.5245)
Supplement: Supplementary file 2 — Figure S1. TBX3 isoform protein structure and functional domains. Figure S2. TBX3 expression in 21NT transfectant and 21MT‐1 transductant cell lines. Figure S3. Functional assessment of TBX3 overexpressing cell lines. Figure S4. Representative images of cell extravasation and invadopodia formation in vivo in the chick chorioallantoic membrane (CAM). Figure S5. TBX3‐mediated invadopodia formation. Figure S6. Effect of TBX3 overexpression on invasiveness in cell lines representing other breast cancer molecular subtypes. Figure S7. Expression of EMT markers with modulation of TBX3 levels. Figure S8. Protein class analysis of direct transcriptional targets of TBX3. Figure S9. Analysis of T‐box binding elements (TBEs) in TBX3‐bound genes identified by ChIP‐array. Figure S10. Subcellular localization of TBX3. Figure S11. H‐scores for TBX3, SLUG, and TWIST1 immunostains in various cell compartments. [file PATH-248-191-s002.pdf]

S1) TBX3 isoform protein structure and functional domains.

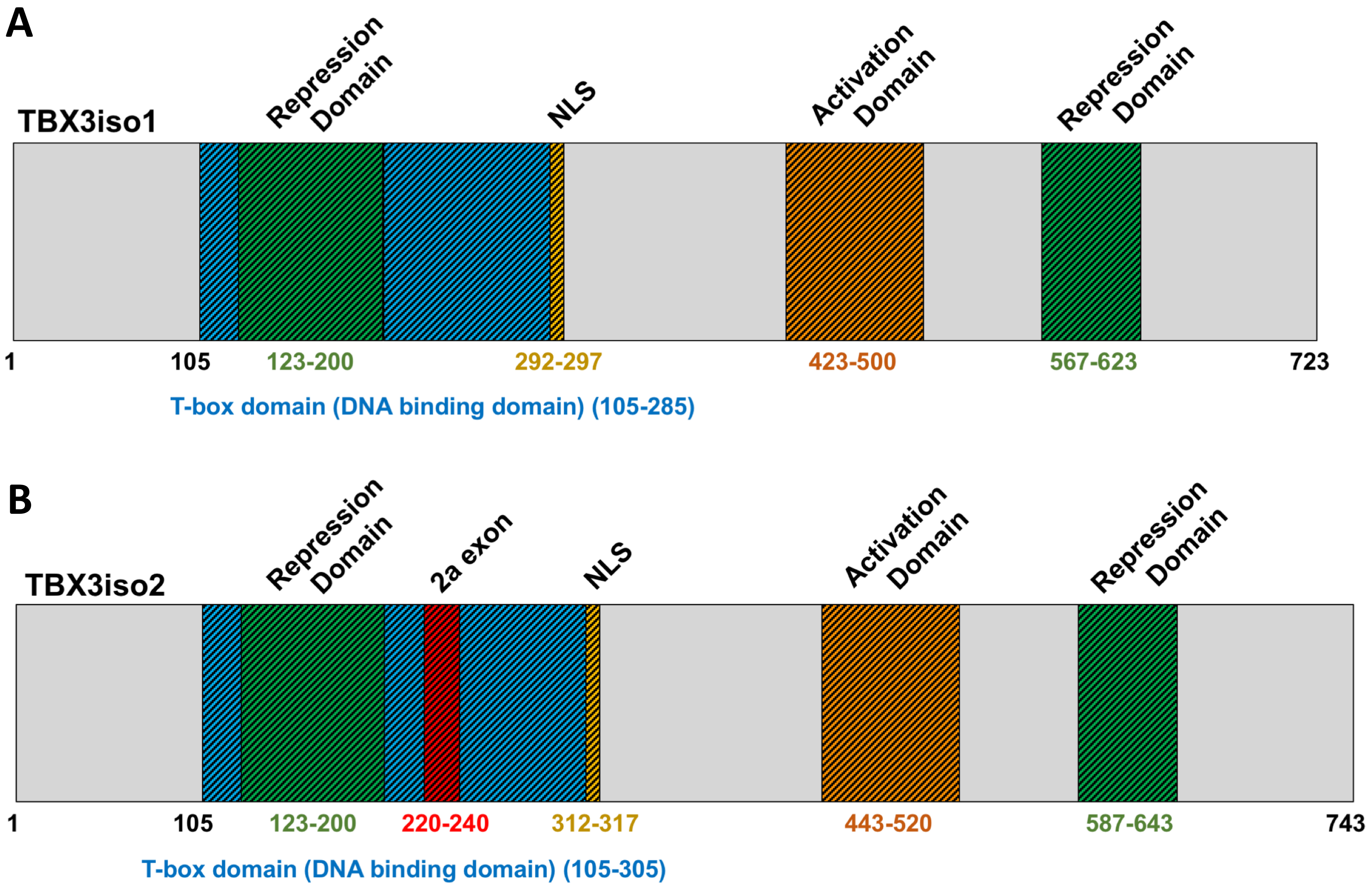

Supplementary Figure S1. TBX3 isoform protein structure and functional domains.

(A-B) Alternative splicing of pre-mRNA gives rise to two TBX3 isoforms: TBX3iso1 **(A)** and TBX3iso2 **(B)**.
The shorter alternatively spliced isoform (TBX3iso1) does not contain the 2a exon, and is 723 amino acids in length. The full-length alternatively spliced isoform (TBX3iso2) contains the 2a exon, and is 743 amino acids in length. The various functional protein domains and locations are shown.

S2) TBX3 expression in 21NT transfectant and 21MT-1 transductant cell lines.

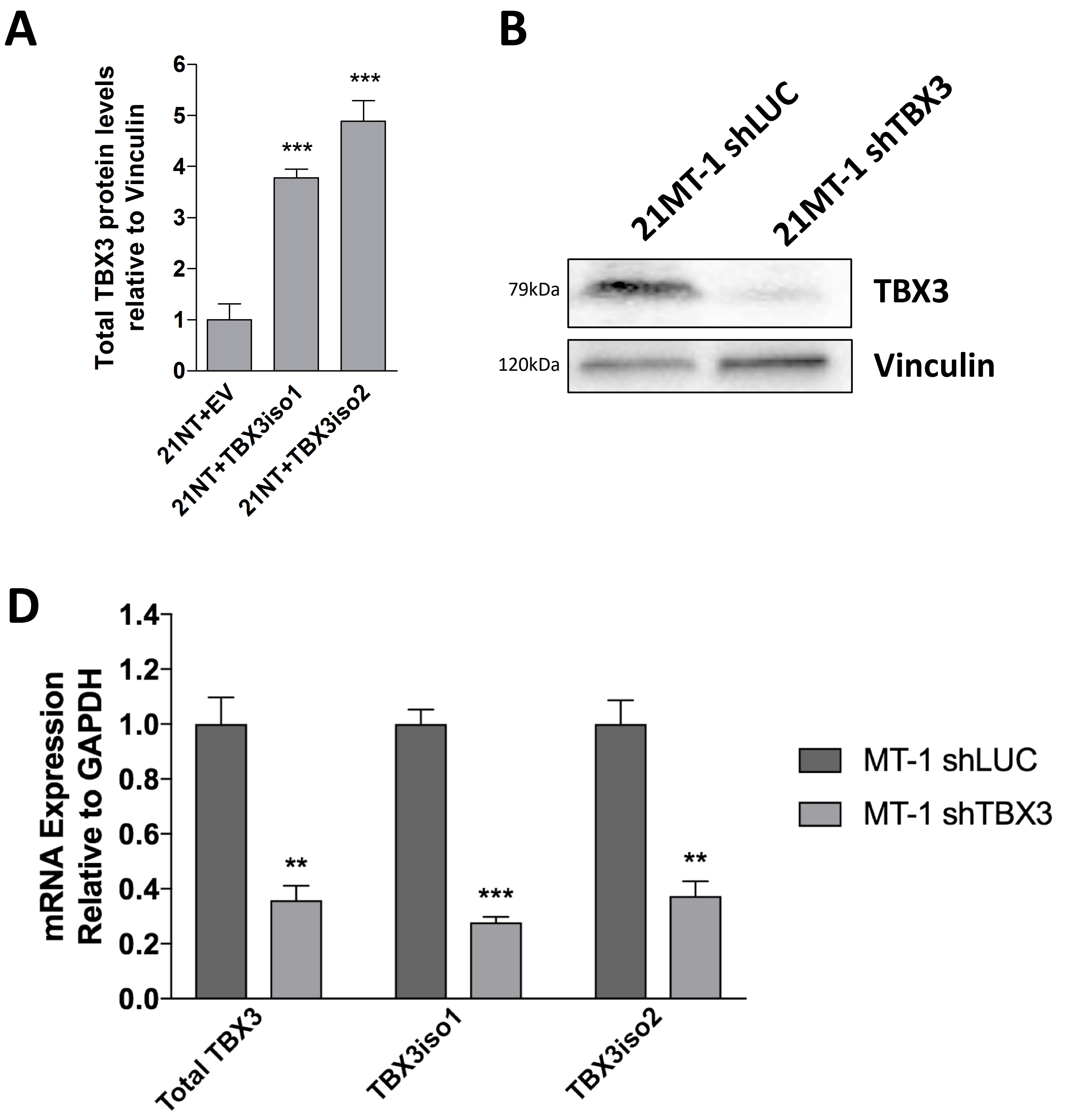

Supplementary Figure S2. TBX3 expression in 21NT transfectant and 21MT-1 transductant cell lines.

(A) Western blot analysis showing total TBX3 protein levels in 21NT stably transfected cells. 21NT cells were transfected to overexpress TBX3iso1 or TBX3iso2, or transfected with an empty vector (EV) control. Protein samples were separated by 10% SDS-PAGE and quantified by densitometry. Protein levels were normalized to Vinculin, which served as the loading control. **(B-C)** Western blot analysis showing total TBX3 protein levels in 21MT-1 stably transduced cells. 21MT-1 cells were transduced with either shLUC (luciferase; off-target control) or shTBX3 which targets both TBX3 isoforms. Protein samples were separated by 10% SDS-PAGE and quantified by densitometry. Protein levels were normalized to Vinculin, which served as the loading control. **(D)** Total TBX3, TBX3iso1, and TBX3iso2 transcript levels were assessed by qRT-PCR in 21MT-1 transduced cells, normalized to GAPDH expression levels, and depicted as fold change relative to the shLUC control. Means derived from three biological replicates were used during analysis.

**p<0.05, **p<0.01, ***p<0.001 by one-way ANOVA with Tukey post-hoc for comparison between three groups, and student's T-test for comparison between two groups.*

S3) Functional assessment of TBX3 overexpressing cell lines.

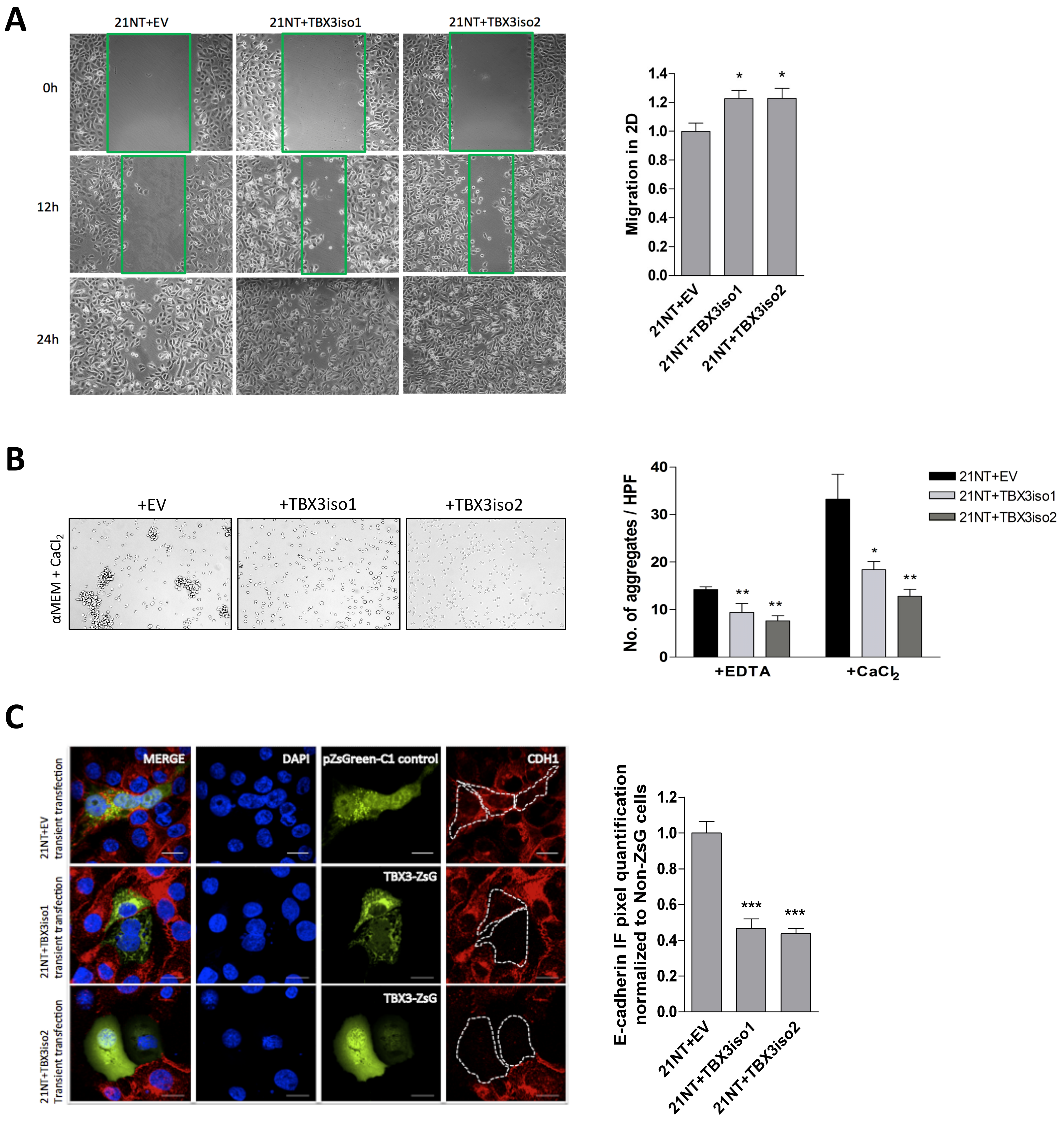

Supplementary Figure S3. Functional assessment of TBX3 overexpressing cell lines.

(A) Assessment of migration in 2D in 21NT transfectant cell lines. A scratch was produced in a confluent monolayer of cells, and migration of cells into the scratch area was monitored at 0, 12 and 24 hours post-scratch. Migration was quantified as ratio of wound area filled at 0 and 12 hours, and normalized to the empty vector control. **(B)** Cell-cell adhesion assay. Cells were harvested and re-suspended in media containing either 3mM EDTA or 1mM $CaCl_2$ and incubated in a petri dish at 37ºC for 30 minutes with gentle agitation. Clusters containing over 4 cells were counted across 10 fields of view. **(C)** 21NT cells were transiently transfected with either a TBX3iso1 or TBX3iso2 plasmid containing a ZsGreen reporter, or empty vector control expressing ZsGreen. CDH1 was visualized using a red-fluorescently labeled antibody. CDH1 signal was quantified by counting pixels within the red channel of ZsGreen positive transfected cells.

**p<0.05, **p<0.01, ***p<0.001 by one-way ANOVA with Tukey post-hoc for comparison between three groups.*

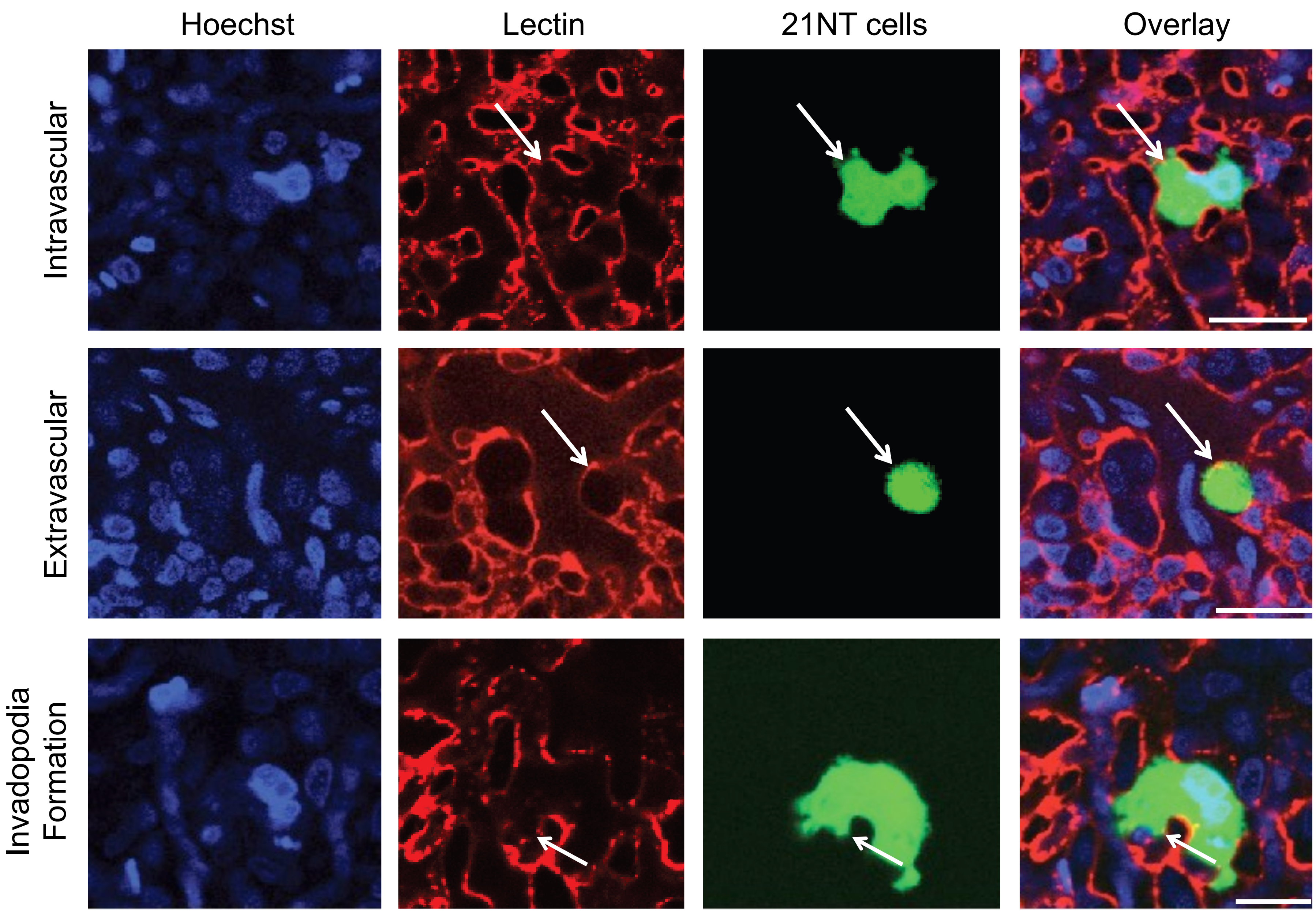

Supplementary Figure S4. Representative images of cell extravasation and invadopodia formation *in vivo* in the chick chorioallantoic membrane (CAM).

Endothelial cells were labelled with rhodamine lectin (red), nuclei were stained with Hoechst (blue) and 21NT cells were labeled with CellTracker Green (green). Representative images of an intravascular cell (top row, white arrow) and extravasated, extravascular, cell (middle row, white arrow) are shown. Invadopodia protrusion were visualized as cell protrusions (bottom row, white arrow) extending through the endothelial layer (red). Cells were imaged in the capillary bed of the CAM using confocal microscopy.

S5) TBX3-mediated invadopodia formation.

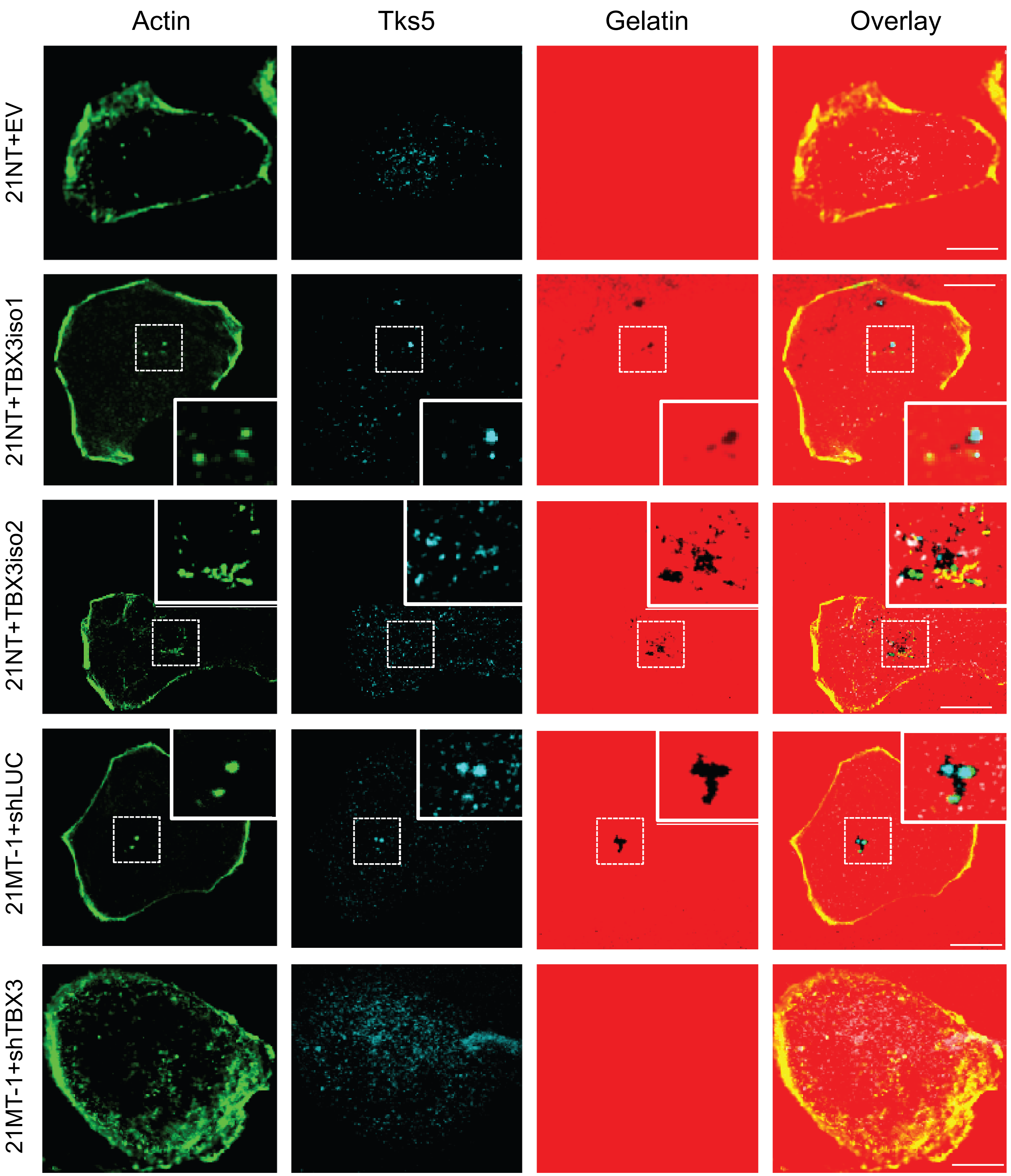

Supplementary Figure S5. TBX3-mediated invadopodia formation.

Cells were plated on gelatin-coated coverslips, fixed, permeabilized, and stained using an anti-TKS5 antibody, followed by Alexa647-conjugated secondary antibody and Alexa488-phalloidin to stain F-actin. Single confocal slices of the ventral surface of cells are shown. Invadopodia were identified as areas of Tks5 (cyan) and actin (green) co-localization overlaying areas of degradation (black holes), as shown in the overlay.

S6) Effect of TBX3 overexpression on invasiveness in cell lines representing other breast cancer molecular subtypes.

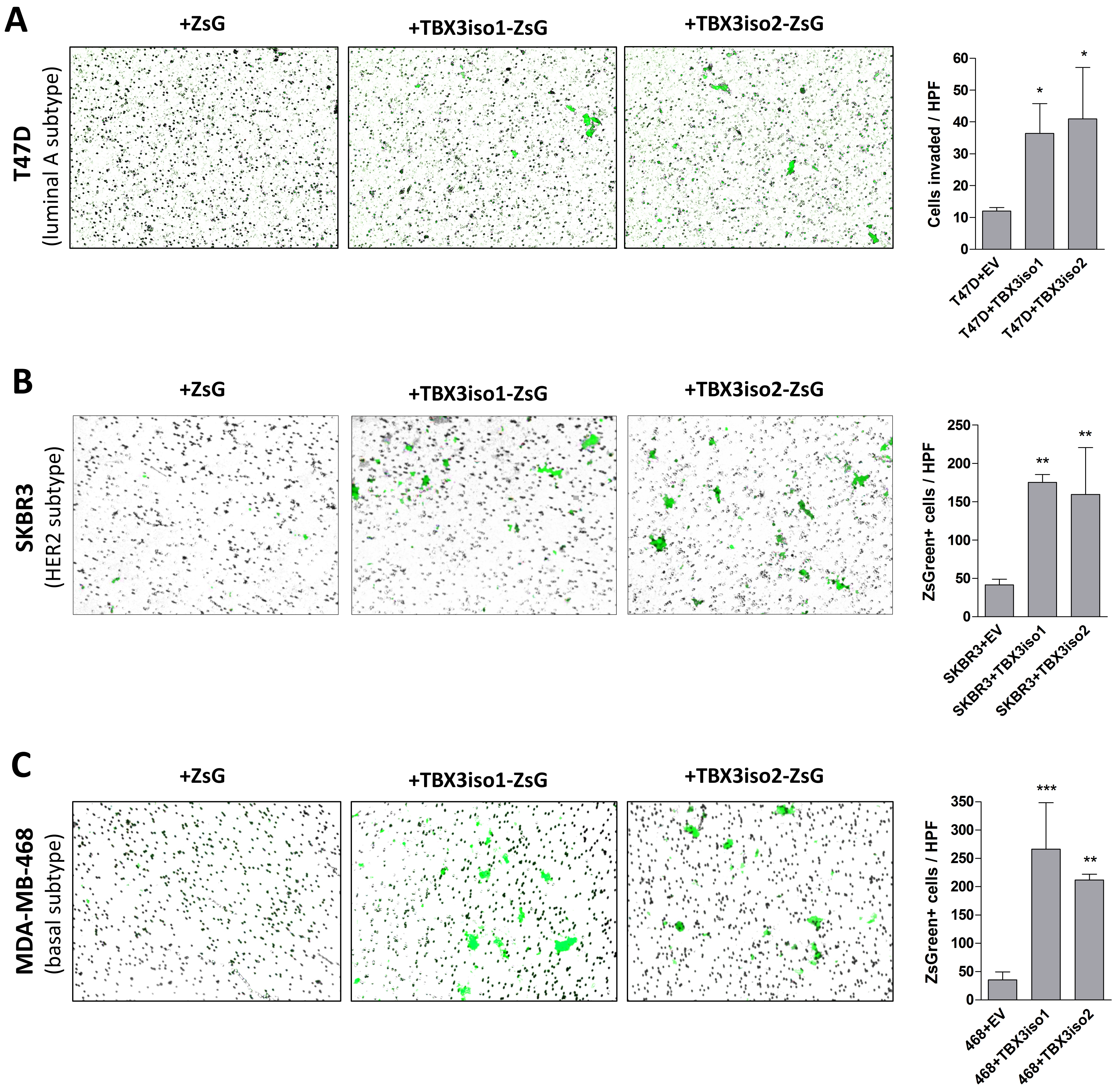

Supplementary Figure S6. Effect of TBX3 overexpression on invasiveness in cell lines representing other breast cancer molecular subtypes.

The invasive ability of TBX3 transfectant lines was assessed using Matrigel-coated transwell inserts. **(A-C)** T-47D (luminal A), SKBR3 (HER2-enriched), and MDA-MB-468 (basal-like) cells were transfected with either an empty vector (EV), or TBX3iso1, or TBX3iso2 construct within the pZsGreen1-C1 vector containing a ZsGreen reporter. Twenty-four hours post-transfection, 50,000 cells were added to the upper chamber of an 8.0μm pore transwell insert coated with Matrigel and allowed to invade for 18 hours. The number of green cells per field of view was used in the analysis.

**p<0.05, **p<0.01, ***p<0.001 by one-way ANOVA with Tukey post-hoc for comparison between three groups.*

S7) Expression of EMT markers with modulation of TBX3 levels.

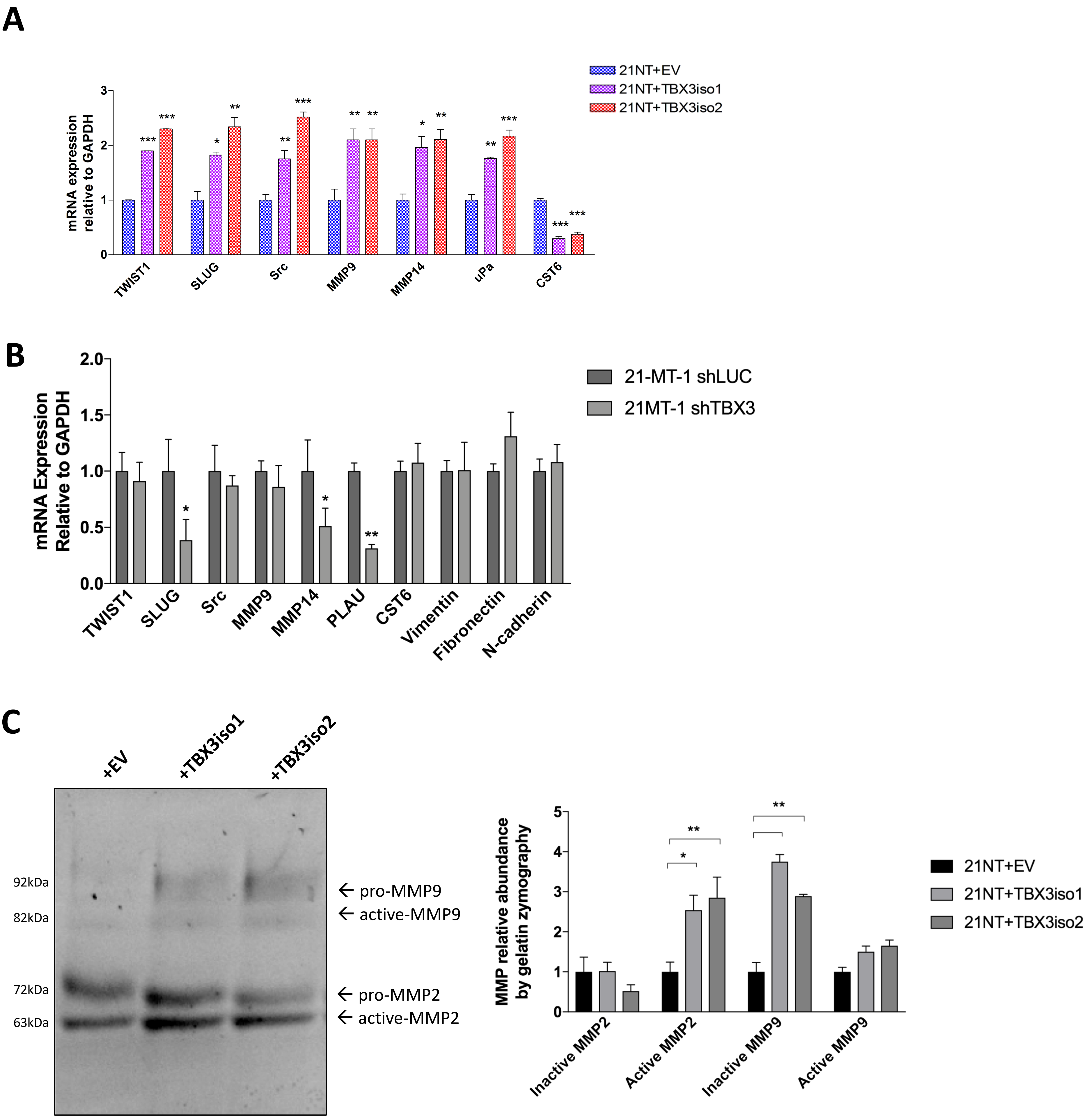

Supplementary Figure S7. Expression of EMT markers with modulation of TBX3 levels.

(A) Expression of several EMT markers was evaluated by qRT-PCR in 21NT transfectants. Expression was normalized to GAPDH expression levels, and depicted as fold change relative to the empty vector control. **(B)** Expression of several EMT markers was evaluated by qRT-PCR in 21MT-1 shLUC (luciferase off-target control) and shTBX3 cell lines. Expression was normalized to GAPDH expression levels, and depicted as fold change relative to the shLUC control. **(C)** Conditioned media was concentrated and resolved on a 10% zymogram gelatin gel. The gel was renatured and developed, and size of the proteolyzed bands was quantified by densitometry using the reverse image (as shown). Active (82 kDa) vs inactive (pro-) MMP9 (92 kDa), and active (63 kDa) vs inactive (pro-) MMP2 (72 kDa) were identified by molecular weight.

**p<0.05, **p<0.01, ***p<0.001 by one-way ANOVA with Tukey post-hoc for comparison between three groups.*

S8) Protein class analysis of direct transcriptional targets of TBX3.

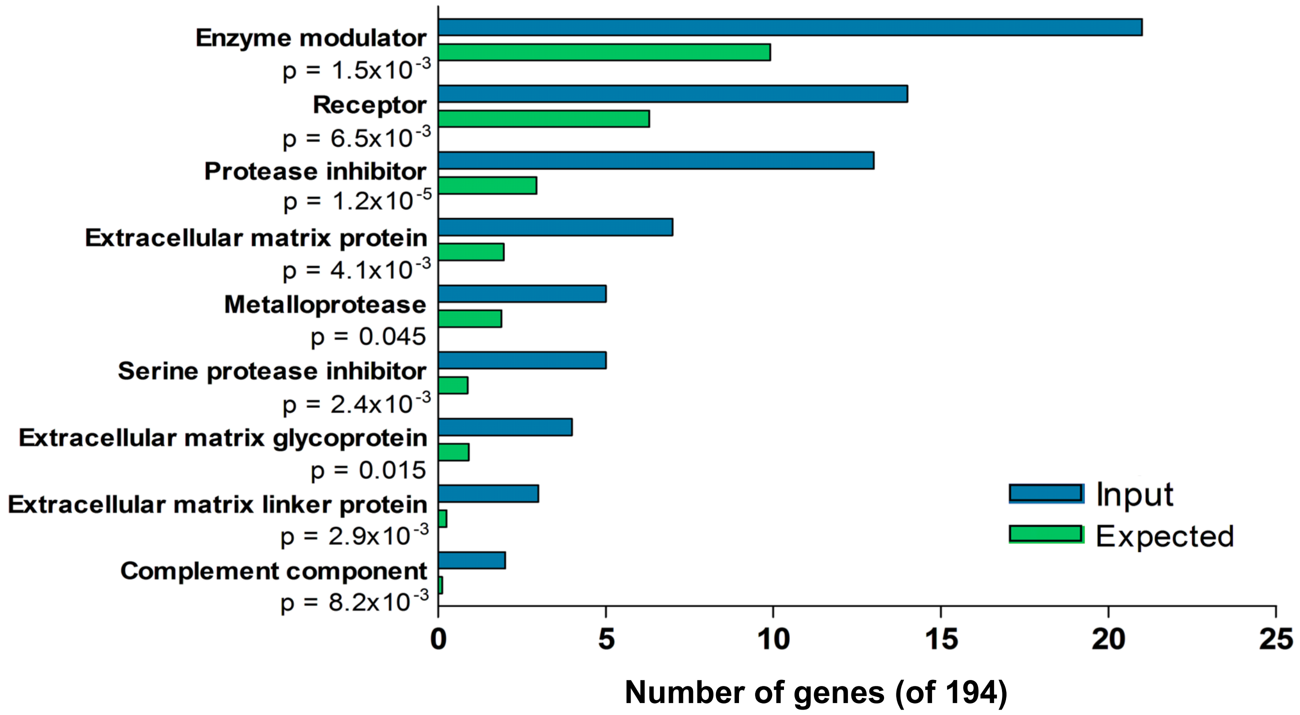

Supplementary Figure S8. Protein class analysis of direct transcriptional targets of TBX3.

The 194 genes directly bound by both TBX3 isoforms in ChIP-array data and whose transcript levels were significantly altered in expression by RNA-Seq (>1.5 fold up or down, FDR<0.05) were analyzed using the PANTHER database, conducting over-representation analysis and focusing on protein class. Protein classes with the lowest p-values are shown. P-values were calculated by comparing expected levels compared to input numbers in gene list.

S9) Analysis of T-box binding elements (TBEs) in TBX3-bound genes identified by ChIP-array.

A

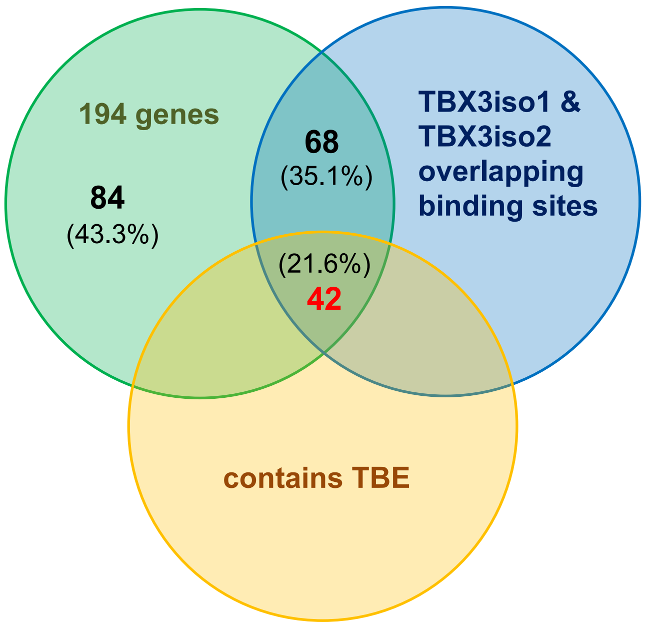

Genes with overlapping TBX3iso1/TBX3iso2 binding sites in ChIP-array and contain TBE (42)

ACSS3	LOC100133669
AGT	OVOL1
ALDH5A1	PADI1
ATP8A1	PAPLN
BBC3	PLEKHA6
CHSY3	PMEPA1
CRISP3	PREX1
CTSH	PTAFR
DYNC1I2	RBM47
FBXO32	SLC11A2
FBXO46	SLC9A2
FGFBP1	SMIM14
G0S2	SNAI2
GGT5	SPON2
GRAMD2	STRA6
HMGCS2	SZT2
HTR3A	TRIM16L
KLF15	TUBB6
LAMB2	UNC5CL
LANCL2	ZNF608
LIMCH1	ZNF615

B

```
Human        GTCCTTGGAGGAGGTGTCAGATGGAGGAGG
Bonobo       GTCCTTGGAGGAGGTGTCAGATGGAGGAGG
Elephant     GTCCTTGGAGGAGGTGTCAGATGGAGGAGG
Dog          GTCCTTGGATGAGGTGTCAGATGGAGGAGG
Wild boar    GTCCTTGGATGAGGTGTCGGATGGAGGAGG
Sea otter    GTCCTTGGACGAAGTGTCAGATGGAGGAGG
```

T-box motif

C

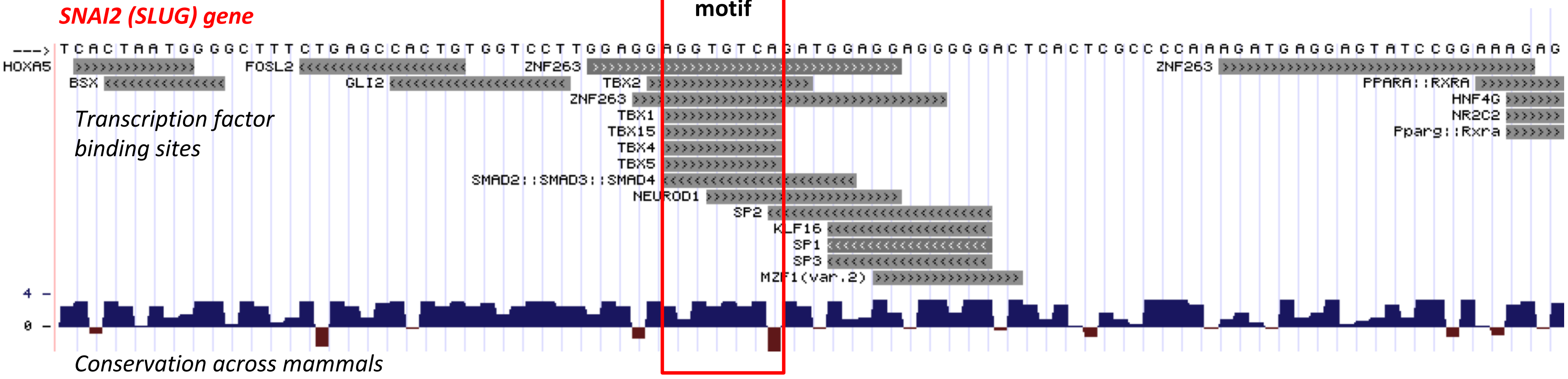

Supplementary Figure S9. Analysis of T-box binding elements (TBEs) in TBX3-bound genes identified by ChIP-array.

(A) Assessment of coupled RNA-Seq and ChIP-array binding sites. A list of 194 genes was obtained in which both TBX3 isoforms were bound to in ChIP-array datasets, and whose transcript levels were significantly altered in expression by RNA-Seq (>1.5 fold up or down, FDR<0.05). Of this list, TBX3iso1 and TBX3iso2 bound the same genomic locations in 110 genes; 42 genes contained an identified TBE, while 68 genes did not contain a TBE. The list of 42 genes which were bound by both TBX3 isoforms in overlapping genomic sites, are significantly altered in expression, and contain a TBE are shown. **(B-C)** Assessment of conservation of T-box binding element (TBE) in SLUG (*SNAI2*) gene across species. Transcription factor binding sites and sequence conservation across mammals are shown in (C) from UCSC Genome Browser. The T-box motif is signified by the red box.

S10) Subcellular localization of TBX3.

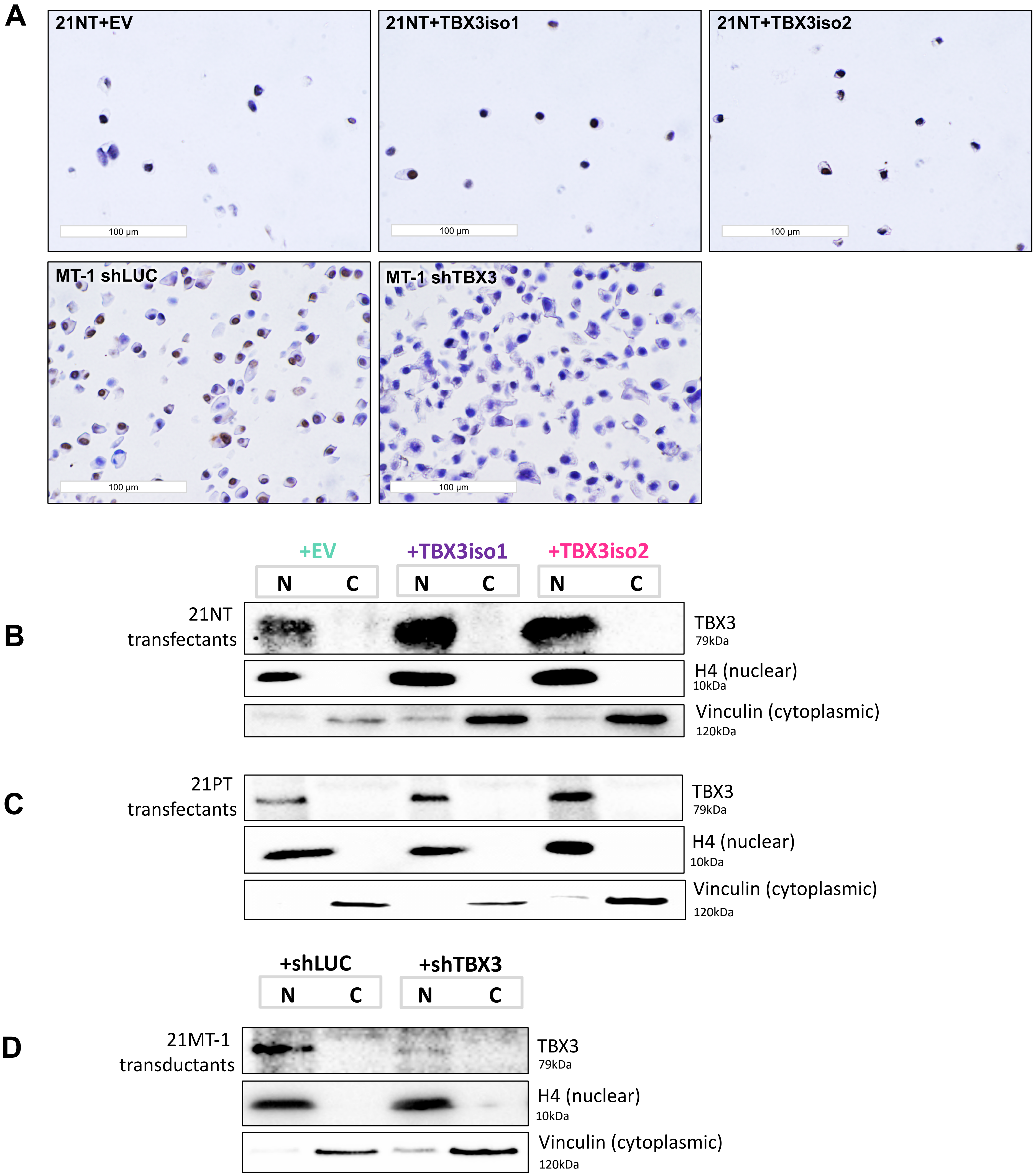

Supplementary Figure S10. Subcellular localization of TBX3.

(A) Cell pellets were prepared, embedded in agarose, and processed to paraffin. Sections were immunostained using anti-TBX3 antibody and counter-stained with Hematoxylin. **(B-D)** Subcellular fractionation was conducted in order to obtain nuclear (N) and cytoplasmic (C) fractions for 21NT transfectant, 21PT transfectant, and 21MT-1 transductant (shLUC; luciferase off-target control; shTBX3 knockdown) cell lines. Subcellular fractions were separated by 10% SDS-PAGE, and western blotting was conducted to assess TBX3 localization in nuclear (histone H4) and cytoplasmic (vinculin) fractions.

S11) H-scores for TBX3, SLUG, and TWIST1 immunostains in various cellular compartments.

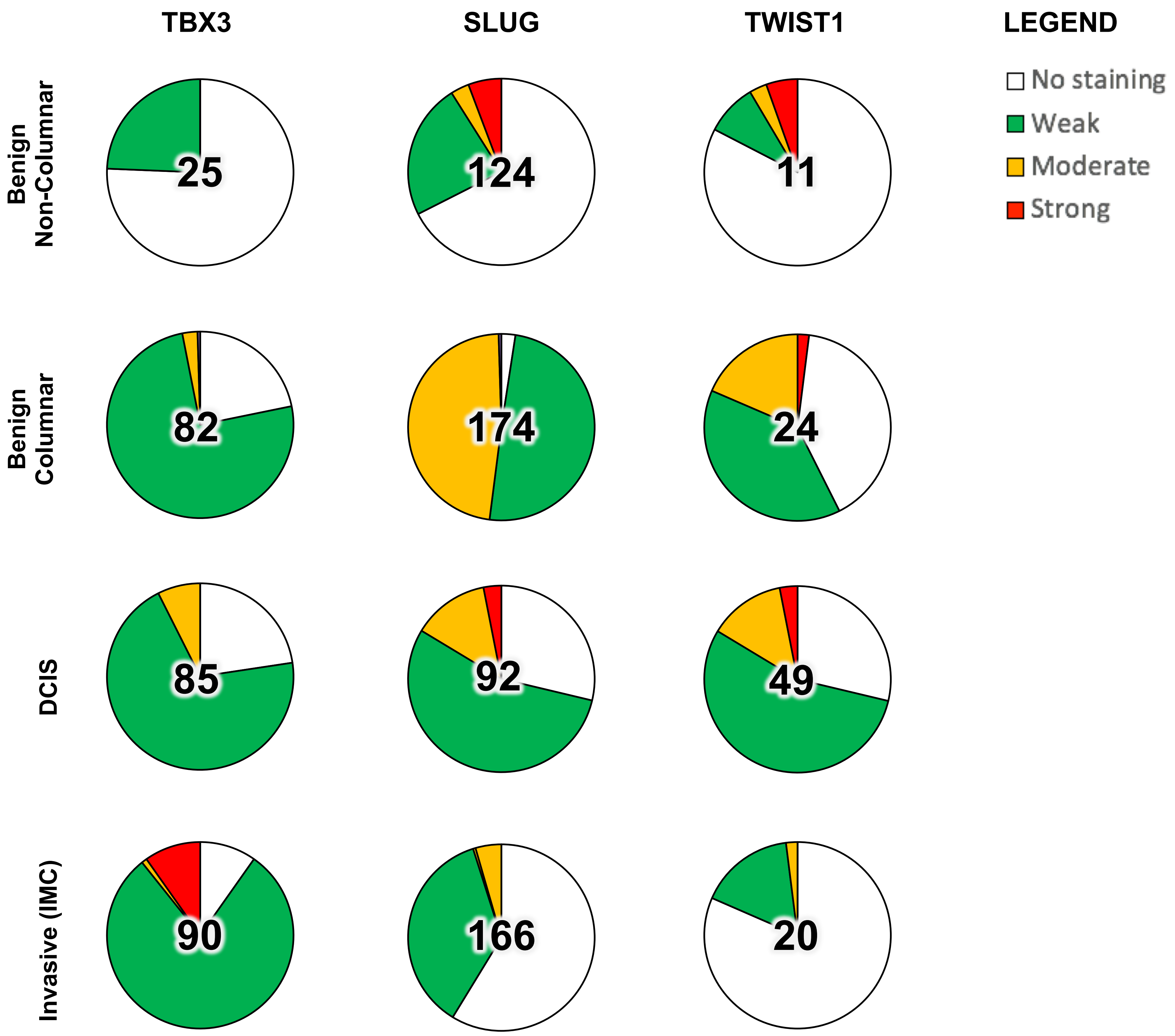

Supplementary Figure S11. H-scores for TBX3, SLUG, and TWIST1 immunostains in various cell compartments.

H-scores of representative slides for each marker and lesion type are shown. Pie charts shown breakdown of staining intensities for each marker, depicting no staining (white), weak staining (green), moderate staining (yellow), and strong staining (red).
