# Supplementary material for: TBX3 promotes progression of pre‐invasive breast cancer cells by inducing EMT and directly up‐regulating SLUG
Source: J Pathol. 2019 Apr 8;248(2):191–203. doi: 10.1002/path.5245 (PMC6593675; doi:10.1002/path.5245)
Supplement: Supplementary file 4 — Table S2. Primer sequences utilized for RT‐qPCR in mRNA studies (mentioned in the supplementary material, Supplementary materials and methods) Table S3. Primer sequences utilized for ChIP‐qPCR validation studies (mentioned in the supplementary material, Supplementary materials and methods) Table S4. Publicly available datasets utilized for analysis (mentioned in the supplementary material, Supplementary materials and methods) [file PATH-248-191-s004.docx]

**TBX3 promotes progression of pre-invasive breast cancer cells by inducing EMT and directly up-regulating SLUG**

Krstic M *et al*. *J Pathol* DOI: 10.1002/path.5245

**Table S2.** Primer sequences utilized for qRT-PCR in mRNA studies

| **mRNA probe** | **(F) Forward and (R) reverse primer sequences, 5' to 3'** |
| --- | --- |
| **Total TBX3** | F: CGCTGTGACTGCATACCAGA R: GTGTCCCGGAAACCTTTTGC |
| **TBX3iso1** | F: AGTGGATGTCCAAAGTCGTCAC  R: CATGGAGTTCAATATAGTAAATCCATGTTTGTCTG |
| **TBX3iso2** | F: AGTGGATGTCCAAAGTCGTCAC  R: CACTTGGGAAGGCCAAAGTAAATCCATG |
| **CDH2 (N-cadherin)** | F: GACGATCCCAATGCCCTCAA R: ACATGTTGGGTGAAGGGGTG |
| **CST6** | F: TGAGGTCCTTGTGGTTCCCT R: CCCTCGGGGACTTATCACATC |
| **FN1 (fibronectin)** | F: AGGAGAATGGACCTGCAAGC R: GAAGTGCAAGTGATGCGTCC |
| **GAPDH** | F: AGGCTGGGGCTCATTTGAAG R: CCATCCACAGTCTTCTGGGTG |
| **MMP14** | F: CCAGCAACTTTATGGGGGTG R: GGCCCATAGGTGGGGTTTTT |
| **MMP9** | F: CTTTGAGTCCGGTGGACGAT R: TCGCCAGTACTTCCCATCCT |
| **TWIST1** | F: GCAAGAAGTCGAGCGAAGAT R: GCTCTGCAGCTCCTCGAA |
| **RPLP0** | F: CCTCATATCCGGGGGAATGTG R: GCAGCAGCTGGCACCTTATTG |
| **SLUG** | F: GCCAAACTACAGCGAACTGG R: GATGGGGCTGTATGCTCCTG |
| **SRC** | F: GCACAGGACAGACAGGCTAC R: TCTGACTCCCGTCTGGTGAT |
| **uPa** | F: TCCAAAGGCAGCAATGAACT R: GTGCTGCCCTCCGAATTTCT |
| **VIM (vimentin)** | F: GGACCAGCTAACCAACGA  R: AAGGTCAAGACGTGCCAGAG |

#

**Table S3.** Primer sequences utilized for ChIP-qPCR validation studies

| **DNA probe** | **(F) Forward and (R) reverse primer sequences, 5' to 3'** |
| --- | --- |
| **CDH1 TSS** | F: TCACAGGTGCTTTGCAGTTC R: GTGAACCCTCAGCCAATCAG |
| **CDH1 coding region** | F: AACAGCTGCTTGGTGACGTT R: CAAGCCTGGGAGTTAGGTG |
| **SNAI2** | F: AATGGGGCTTTCTGAGCCAC R: TCCACGCCCAGCTACCCAA |

**Table S4.** Publicly available datasets utilized for analysis

| **FILE** | **SOURCE** | **REFERENCE (or *LINK)*** |
| --- | --- | --- |
| **POLR2A ChIA- PET** | ENCODE:  **ENCSR000CAA** | [*http://hgdownload.cse.ucsc.edu/goldenPath/hg19/encodeDCC/wgEnco*](http://hgdownload.cse.ucsc.edu/goldenPath/hg19/encodeDCC/wgEnco) *deGisChiaPet/* |
| **DNase I hypersensitivity (master list)** | ENCODE:  **wgEncodeAwgDna seMasterSites** | [*http://hgdownload.cse.ucsc.edu/goldenpath/hg19/encodeDCC/wgEncod*](http://hgdownload.cse.ucsc.edu/goldenpath/hg19/encodeDCC/wgEncod) *eAwgDnaseMasterSites/* |
| **TCGA Breast Cancer (BRCA)** | GDC Project ID:  **TCGA-BRCA** | *https://portal.gdc.cancer.gov/projects/TCGA-BRCA* |
| **Farmer Breast** | GEO:  **GSE1561** | Farmer P, Bonnefoi H, Becette V, *et al*. Identification of molecular apocrine breast tumours by microarray analysis. *Oncogene* 2005; **24:** 4660–4671. PMID: 15897907 |
| **Desmedt Breast** | GEO:  **GSE7390** | Desmedt C, Piette F, Loi S, *et al*. Strong time dependence of the 76-gene prognostic signature for node-negative breast cancer patients in the TRANSBIG multicenter independent validation series. *Clin Cancer Res* 2007; **13:** 3207–3214. PMID: 17545524 |
